# Humanization of yeast genes with multiple human orthologs reveals functional divergence between paralogs

**DOI:** 10.1371/journal.pbio.3000627

**Published:** 2020-05-18

**Authors:** Jon M. Laurent, Riddhiman K. Garge, Ashley I. Teufel, Claus O. Wilke, Aashiq H. Kachroo, Edward M. Marcotte

**Affiliations:** 1 Center for Systems and Synthetic Biology, Institute for Cellular and Molecular Biology, The University of Texas at Austin, Austin, Texas, United States of America; 2 Institute for Systems Genetics, NYU Langone Health, New York, New York, United States of America; 3 Department of Molecular Biosciences, The University of Texas at Austin, Austin, Texas, United States of America; 4 Department of Integrative Biology, The University of Texas at Austin, Austin, Texas, United States of America; 5 Santa Fe Institute, Santa Fe, New Mexico, United States of America; 6 The Department of Biology, Centre for Applied Synthetic Biology, Concordia University, Montreal, Quebec, Canada; Universitetet i Bergen, NORWAY

## Abstract

Despite over a billion years of evolutionary divergence, several thousand human genes possess clearly identifiable orthologs in yeast, and many have undergone lineage-specific duplications in one or both lineages. These duplicated genes may have been free to diverge in function since their expansion, and it is unclear how or at what rate ancestral functions are retained or partitioned among co-orthologs between species and within gene families. Thus, in order to investigate how ancestral functions are retained or lost post-duplication, we systematically replaced hundreds of essential yeast genes with their human orthologs from gene families that have undergone lineage-specific duplications, including those with single duplications (1 yeast gene to 2 human genes, 1:2) or higher-order expansions (1:>2) in the human lineage. We observe a variable pattern of replaceability across different ortholog classes, with an obvious trend toward differential replaceability inside gene families, and rarely observe replaceability by all members of a family. We quantify the ability of various properties of the orthologs to predict replaceability, showing that in the case of 1:2 orthologs, replaceability is predicted largely by the divergence and tissue-specific expression of the human co-orthologs, i.e., the human proteins that are less diverged from their yeast counterpart and more ubiquitously expressed across human tissues more often replace their single yeast ortholog. These trends were consistent with in silico simulations demonstrating that when only one ortholog can replace its corresponding yeast equivalent, it tends to be the least diverged of the pair. Replaceability of yeast genes having more than 2 human co-orthologs was marked by retention of orthologous interactions in functional or protein networks as well as by more ancestral subcellular localization. Overall, we performed >400 human gene replaceability assays, revealing 50 new human–yeast complementation pairs, thus opening up avenues to further functionally characterize these human genes in a simplified organismal context.

## Abstract 2

To understand the extent to which ancestral functions are retained among duplicated genes, this study systematically swaps hundreds of essential yeast genes with their human orthologs from expanded gene families. The results reveal widespread differential replaceability within assayed families, and 50 new human-yeast complementation pairs.

## Introduction

**Fig 1:**
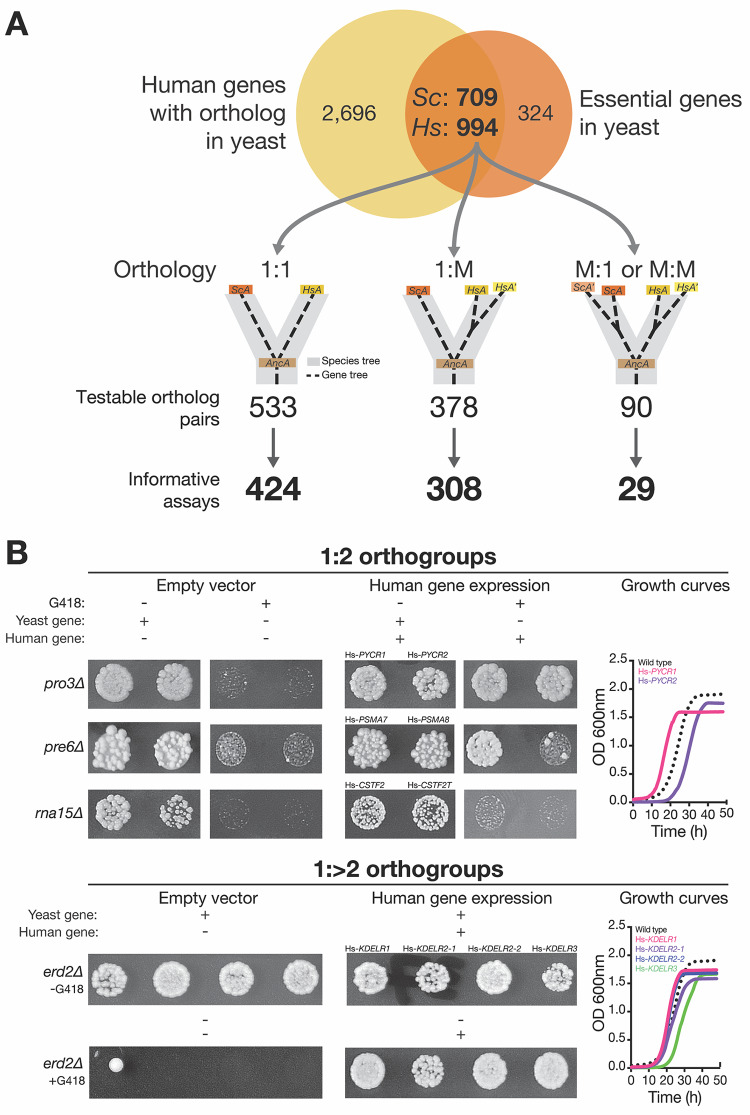
Systematic functional replacement of essential yeast genes with multiple human co-orthologs. (A) We identified 994 human genes that are orthologs of 709 essential yeast genes. Of these ortholog pairs, we had previously obtained results for 424 pairs with no duplications in either yeast or human lineage (i.e., with 1:1 orthology) [5]. In this study, we tested the remaining set of essential yeast genes that have acquired lineage-specific duplications, classifying them as 1:M (1 yeast to 2 or more human co-orthologs) or M:M (≥2 yeast to ≥2 human co-orthologs) or M:1 (≥2 yeast to 1 human ortholog). There are 140 essential yeast genes with more than one human ortholog, representing 378 ortholog pairs to be tested. In the case of the 1:M category, we obtained 308 informative assays out of 378 testable pairs, whereas in the case of the M:M or M:1 set, we had 29 informative assays out of 90 testable pairs. Replaceability assays were performed in both the hetKO collection and the temperature-sensitive haploid yeast collection. (B) Representative assays performed in yeast hetKO strains for 1:2 (top) and 1:>2 (bottom) are shown. HetKO yeast strains expressing human genes were sporulated, and the sporulation mix was spotted on Magic Marker medium (see Materials and methods) with (yeast gene absent) or without (yeast gene present) G418. Assays were performed with empty vector control (human gene absent) or yeast expression vectors carrying a human cDNA (human gene expression). In the 1:2 class, three different outcomes of human gene replaceability in yeast were obtained. Top panel: both human co-orthologs (Hs-*PYCR1* and Hs-*PYCR2*) can replace their yeast equivalent (Sc-*PRO3*). Middle panel: one of the two human co-orthologs (Hs-*PSMA7* but not Hs-*PSMA8*) can replace its yeast equivalent (Sc-*PRE6*). Bottom panel: neither of the two human co-orthologs (Hs-*CSTF2* or Hs-*CSTF2T*) can replace its yeast equivalent (Sc-*RNA15*). In the 1:>2 class, an example of all human co-orthologs (Hs-*KDELR1*, two variants of Hs-*KDELR2*, and Hs-*KDELR3*) replacing their yeast gene counterpart Sc-*ERD2* equally well is shown. The yeast growth assays for these replaceable human genes are shown on the right for each. Haploid yeast gene deletion strains carrying plasmids expressing functionally replacing human genes (colored solid lines) generally exhibit comparable growth rates to the wild-type parental yeast strain BY4741 (black dotted lines). Plotted growth curves display the mean of triplicate growth experiments. 1:M, one-to-many; *CSTF2*, Cleavage stimulating factor subunit 2; *CSTF2T*, Cleavage stimulating factor subunit 2 tau subunit; *ERD2*, ER lumen protein-retaining receptor, endoplasmic reticulum retention defective 2; hetKO, heterozygous diploid knockout; Hs, *H*. *sapiens*;; HsA, *H*. *sapiens* co-ortholog A; *KDELR1*, ER lumen protein-retaining receptor 1; *KDELR2*, ER lumen protein-retaining receptor 2; *KDELR3*, ER lumen protein-retaining receptor 3; M:1, many-to-one; M:M, many-to-many; OD, optical density; *PRE6*, Proteasome subunit alpha type-4; *PRO3*, Delta 1-pyrroline-5-carboxylate reductase; *PSMA7*, Proteasome subunit alpha type-7; *PSMA8*, Proteasome subunit alpha type-8; *PYCR1*, Pyrroline-5-carboxylate reductase-1; *PYCR2*, Pyrroline-5-carboxylate reductase-2; Sc, *S*. *cerevisiae*; ScA, *S*. *cerevisiae* ortholog A.

Humans and budding yeast differ dramatically with respect to cell and tissue organization, metabolism, motility, and environment; nonetheless, yeast has remained an important eukaryotic model for informing and answering questions related to our own biology. Thousands of human genes have clearly identifiable homologs in yeast [1] **(Fig 1A**), all deriving from genes of a common opisthokont ancestor living approximately a billion years ago [2]. Many studies have related function and disruption of yeast genes to their human counterparts, relying on a strong tendency for ancestral function to be retained between homologous genes (i.e., for sequence similarity to imply functional similarity). A more rigorous test of ancestral function being retained involves exchanging genes between related species to test for their ability to replace loss of the homologous genes. Indeed, interspecies swaps have been used for decades to identify homologs with equivalent function even before sequence comparison was as routine as it is today [3, 4]. More recently, large-scale systematic studies have identified many human genes that can substitute for their yeast equivalents and sustain yeast growth [5–8]. These systematic studies of functional replacement of yeast genes by homologs from humans [5–8] and bacteria [9] demonstrate the power of interspecies gene swaps to directly test functional divergence and identify properties that determine functional conservation across vast evolutionary distances.

Most (but not all) prior yeast humanization studies have considered genes that have not acquired observable duplications in either the human or yeast lineage, referred to as 1:1 orthologs; that is, one yeast gene has one human ortholog. (Two homologous genes related by speciation are termed “orthologs” and are distinguished from those related by gene duplication, termed “paralogs” [10, 11].) However, many human genes in particular belong to gene families that have duplicated and diverged along the human lineage. Importantly, an expanded family of human genes may often correspond to a single gene on the yeast lineage (or vice versa). (For simplicity, we will generally refer to such cases as co-orthologs, although lineage-specific gene deletions can complicate such assignments.)

More broadly, gene duplication is regarded as a major contributor to the production of new genetic material [12]. Because of the immediate functional redundancy and dosage increase created following duplication, a common fate of duplicated genes is loss of one functional copy, returning to the ancestral state [13]. However, duplicate genes can also either retain their ancestral roles or diverge and adopt new functions [10, 11]. Lineage-specific duplications also have a major practical impact on how homologous genes between species are identified and defined. How ancestral functions are partitioned, lost, or retained during these duplication events is a major topic of study for evolutionary biology [14–19]. Thus, expanding systematic tests of functional replacement across these expanded gene families could help address questions of how functions are retained or lost following duplication, as well as provide researchers with many more opportunities to study human gene function in the simplified context of budding yeast.

In this work, we sought to directly assay functional divergence within expanded gene families by using yeast humanization. We identified all essential yeast genes in yeast human ortholog groups (orthogroups) that have undergone expansions in the human and/or yeast lineage and systematically replaced the yeast orthologs with each of their human co-orthologs, assaying functional replaceability by complementation of a lethal growth defect. We find that duplicated human genes tend to differentially replace their yeast ortholog, rarely observing broad replaceability across members of expanded human gene families. Further, we quantified the ability of several protein-, gene-, or ortholog-based properties to explain the differential ability of human co-orthologs to replace and further support our observations with in silico simulations of protein evolution post-duplication.

Collectively, our results suggest that within paralogous human gene families, at least one gene generally tended to retain ancestral function well enough to replace a billion-year-diverged ortholog in a yeast cell. These resulting strains and data serve as important resources for addressing questions in evolutionary biology, as well as for genetic and biotechnology applications. For example, they will enable new yeast-based drug screens against human genes, guide pathway and genome engineering efforts, and enable tests of human disease allele functionality, among other applications, all in the much simpler eukaryotic context of the yeast cell [20]. Our data underscore the remarkable extent to which genes are functionally equivalent between humans and yeast, demonstrating the power of distant model organisms for studying human processes.

## Results and discussion

### Identifying and selecting orthologs in expanded orthogroups and ortholog replaceability assays

Previous work from our group demonstrated that roughly half (47%) of one-to-one (1:1) yeast-to-human orthologs were capable of replacing essential yeast genes across a panel of three complementation assays **(Fig 1A)** [5]. We had specifically restricted these earlier tests to ortholog groups for which no lineage-specific gene duplications were easily identified to mitigate any effects of functional redundancy between paralogs. In the present study, we now focus on these cases of ortholog groups that have undergone lineage-specific gene family amplifications **(Fig 1A)**. We again restricted our test set to include only those groups in which the yeast gene was annotated as essential for growth under standard laboratory conditions **(Fig 1A, S1 Table)** [21, 22] and tested each human co-ortholog for its ability to individually complement loss of its yeast ortholog. We tested all human gene:yeast strain combinations for which reagents were available, even when not all members of an orthogroup could be tested.

In total, we identified 2,073 orthogroups, comprising 4,556 ortholog pairs (2,424 yeast proteins and 3,690 human proteins), using InParanoid [1]. Considering only yeast proteins essential for growth in standard laboratory conditions, there are 1,001 ortholog pairs **(Fig 1A)** distributed in 706 orthogroups. In our previous study, we obtained results for 424 human genes belonging to 1:1 orthogroups **(Fig 1A)** [5]. The remaining orthogroups comprise 468 additional pairs with essential yeast orthologs **(Fig 1A)** and are the focus of this study. These expanded protein families were further split according to whether they had expansions only on the human lineage (one-to-many [1:M]) or expansions on either the yeast or both lineages (many-to-one or many-to-many, collectively referred to here as M:M), resulting in 378 1:M pairs (involving 140 orthologous yeast genes) and 90 M:M pairs (representing 36 yeast genes and 83 human genes) **(Fig 1A)**. The 1:M orthogroups varied widely in size, with the majority having only two human members, and the largest orthogroup (the melanoma antigen gene [MAGE] family) comprising 28 human co-orthologs **(S1 Fig)**.

We obtained human gene clones for our assays from either the human ORFeome collection or human sequence-verified Mammalian Gene Collection [23, 24]. Human genes were subcloned via Gateway cloning [25] into yeast expression vectors under the control of the yeast glyceraldehyde-3-phosphate dehydrogenase (GPD) promoter, driving constitutive, robust expression. We performed complementation assays in one or both of two distinct yeast strain backgrounds: The first, referred to as the heterozygous diploid knockout deletion (hetKO) collection, represents a collection of diploid yeast strains, each of which contains a hetKO of a single gene, with a selectable marker cassette allowing isolation of the haploid knockout post-sporulation [26]. The second mutant background is a library of temperature-sensitive (TS) haploid yeast strains, each harboring a mutant allele encoding a TS form of the protein [27] that can be inactivated by growth at restrictive temperatures (typically 36 or 37°C). These two strain backgrounds allow precise conditional loss of an essential yeast gene or protein for assaying complementation by its human ortholog(s) **(Fig 1B, Materials and methods)**. (We had previously demonstrated that 41 of 42 assayed strains could be rescued by plasmid-based expression of the corresponding yeast gene [5, 9], confirming the utility of this approach.) We verified our assay results by isolating haploid humanized yeast gene knockout strains (either using Magic Marker medium or by tetrad dissection) while simultaneously verifying dependency on the human gene-encoding plasmid **(S3 Fig, S1 Table, Materials and methods)**. We further performed quantitative growth assays for each of the humanized yeast strains to more accurately characterize the robustness of complementation. The majority of complementing human genes exhibits similar growth profiles to the parental yeast strain **(Fig 1B, S4 and S5 Figs)**.

In total, we obtained informative results for 308 of the 378 1:M ortholog pairs. (“Informative” here refers to those assays in which all controls behaved appropriately. See **Materials and methods** for details.) This translates to successful results from at least one human ortholog for approximately 93% (130 of 140) of testable 1:M yeast genes. Of the 90 M:M pairs, 29 were successfully assayed. **(Fig 1A, S1 Table)**. Because of the scarcity of replaceable M:M ortholog pairs and the complicated nature of their orthology relationships, we simply report the results of their assays but do not include them in any subsequent analysis.

### Orthologs in expanded human gene families differentially replace their yeast orthologs

**Fig 2:**
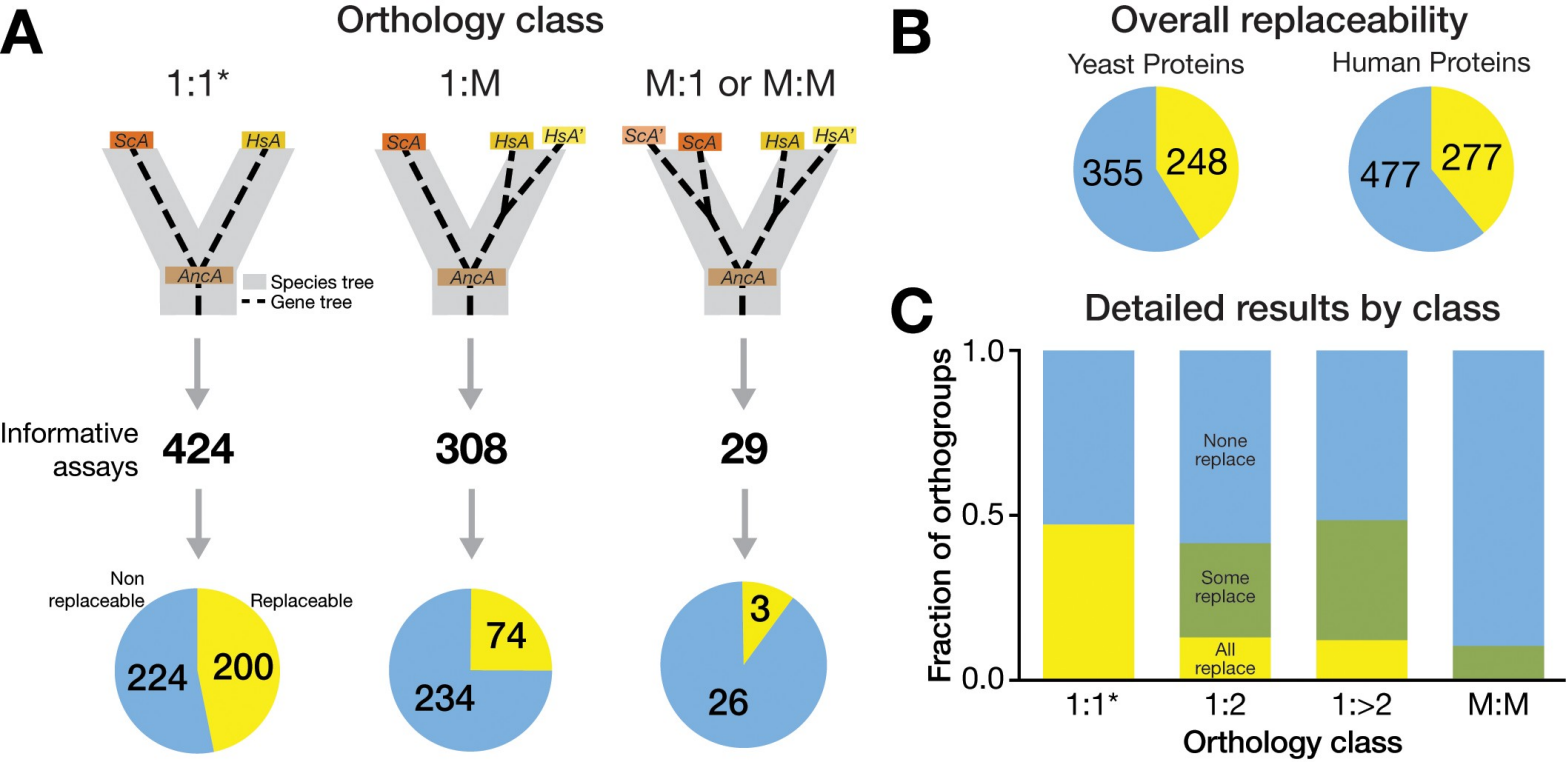
Distribution of replaceability across orthology classes. Only rarely did all human co-orthologs in one orthogroup replace. Rather, a family of human proteins typically had one or a few replaceable members or none at all. (A) Previously, systematic replacement of essential yeast genes with 1:1 orthologs (1 yeast to 1 human) demonstrated nearly 50% replaceability of essential yeast genes [5]. Here, we tested the replaceability of essential yeast genes with their human counterparts that have acquired lineage-specific duplications in either yeast or human lineage. Of the 308 informative assays obtained in the 1:M class (1 yeast to 2 or more human co-orthologs), 74 human genes replaced their yeast equivalents, whereas 234 did not. Of the 29 informative assays obtained in the M:1 and M:M class (≥2 yeast to 1 or more human co-orthologs), three human genes replaced their yeast equivalents, whereas 26 did not. (B) Combining our previous replaceability assays [5] with the assays done in this study, we have identified 248 essential yeast genes that are functionally replaceable by their human counterparts and 355 that are not. From the perspective of human proteins, 277 replace their yeast versions, whereas 477 do not. Summary of all the humanized yeast assays performed thus far. (C) Nearly half of the essential yeast genes belonging to the 1:1 orthology class were replaceable by their human equivalents (yellow). The distribution of essential yeast genes replaced by at least one human ortholog in the 1:M (both 1:2 and 1:>2 combined) closely matched the 1:1 results. These yeast genes were rarely replaced by all human co-orthologs in an orthogroup (yellow), with the majority of replaceability falling in the differentially replaceable set (green). M:M yeast orthologs were rarely replaceable by any human ortholog, with only three (of 29 tested) human genes replacing three separate yeast orthologs. Nonreplaceable genes are indicated in blue. * indicates data from [5]. 1:M, one-to-many; HsA, *H*. *sapiens* co-ortholog A; HsA′, *H*. *sapiens* co-ortholog A′; M:1, many-to-one; M:M, many-to-many; ScA, *S*. *cerevisiae* ortholog A.

Of the 130 successfully tested 1:M yeast genes, 52 (40%) had at least one human ortholog that replaced, whereas 78 were not replaceable by any tested human ortholog **(Fig 2, S4 and S5 Figs, S1 Table)**. Notably, this rate of replaceability from the yeast gene perspective is similar to that previously observed for 1:1 orthologs (40% compared with 47%) [5].

From the perspective of human genes in these expanded gene families, 74 of the 308 successfully tested 1:M human genes could functionally replace their yeast ortholog, whereas 234 could not **(Fig 2A, S4 and S5 Figs)**. Of the orthogroups with at least one complementing human gene, the majority (34/52 groups, or 65%) showed differential replaceability, whereas all human co-orthologs in a group replaced only in a few cases (12/52 or approximately 23%) **(Fig 2C, S4 and S5 Figs, S1 Table)**. In marked contrast, the great majority of human genes belonging to the M:M class were not able to replace their yeast orthologs, with only three human genes replacing in these cases, distributed across three different ortholog groups **(Fig 2C, S4C Fig, S1 Table)**.

When available, we compared our 1:M and M:M replaceability results with published reports, observing a strong agreement, with our assays recapitulating literature results (here, defined solely by the YeastMine database [28]) with 88% accuracy (**S2 Table**). Accounting for our previous assays as well as other published tests, we report an additional 50 novel yeast–human complementation pairs. With previously published cases from the 1:1 set, this brings our total count of known yeast genes with swappable human orthologs to 280 [5] **(Fig 2B)**.

### Computational analysis of trends governing replaceability

Because the ability of human co-orthologs to functionally replace their singleton yeast orthologs was generally differential within expanded gene families, we sought to identify characteristic features governing selective replaceability of specific human genes in expanded orthogroups. To that end, we assembled and/or calculated a number of quantitative properties for all genes and ortholog pairs. These properties include direct properties of the genes in an orthogroup (e.g., protein length, codon usage bias), as well as comparative properties between co-orthologs (e.g., protein sequence similarity, length difference). We could then assess each property for its ability to explain replaceability as the area under a receiver operating characteristic (ROC) curve, treating each feature as an individual classifier, in a similar manner to the previous 1:1 ortholog study [5]. To simplify pairwise comparisons, we considered median values of co-orthologs within each orthogroup that had the same replaceability status (i.e., in which both could replace or not) **(S6 Fig)**. Our analysis is robust to missing data, ignoring features for gene pairs that had no informative assay. Of the 378 testable 1:M pairs, the majority (208 pairs in 104 orthogroups) belonged to a group that had only two human co-orthologs (referred to as the 1:2 class) **(S1 Fig)**. The remaining orthogroups with more than two human co-ortholog members were dubbed the 1:>2 class. Owing to this disparity, we chose to consider these two groups separately in subsequent analyses.

### Differential replaceability in 1:2 orthogroups is predicted by co-ortholog divergence and mRNA expression specificity

In the 104 1:2 ortholog groups, we obtained informative results for 171/208 human genes (94/104 yeast genes) **(S1 and S4 Figs, S1 Table)**. Forty-five of the tested human genes functionally replaced the yeast gene, whereas 127 could not (36 human genes were either not tested or did not result in an informative assay). Seventy-seven of these groups were completely tested (i.e., both human co-orthologs were assayed and yielded informative results), 17 groups had only a single tested human gene, and 10 had no informative assay results (**S1 Table**). From the yeast genes’ perspectives, 34/95 (36%) of those assayed were replaceable by at least one human ortholog.

**Fig 3:**
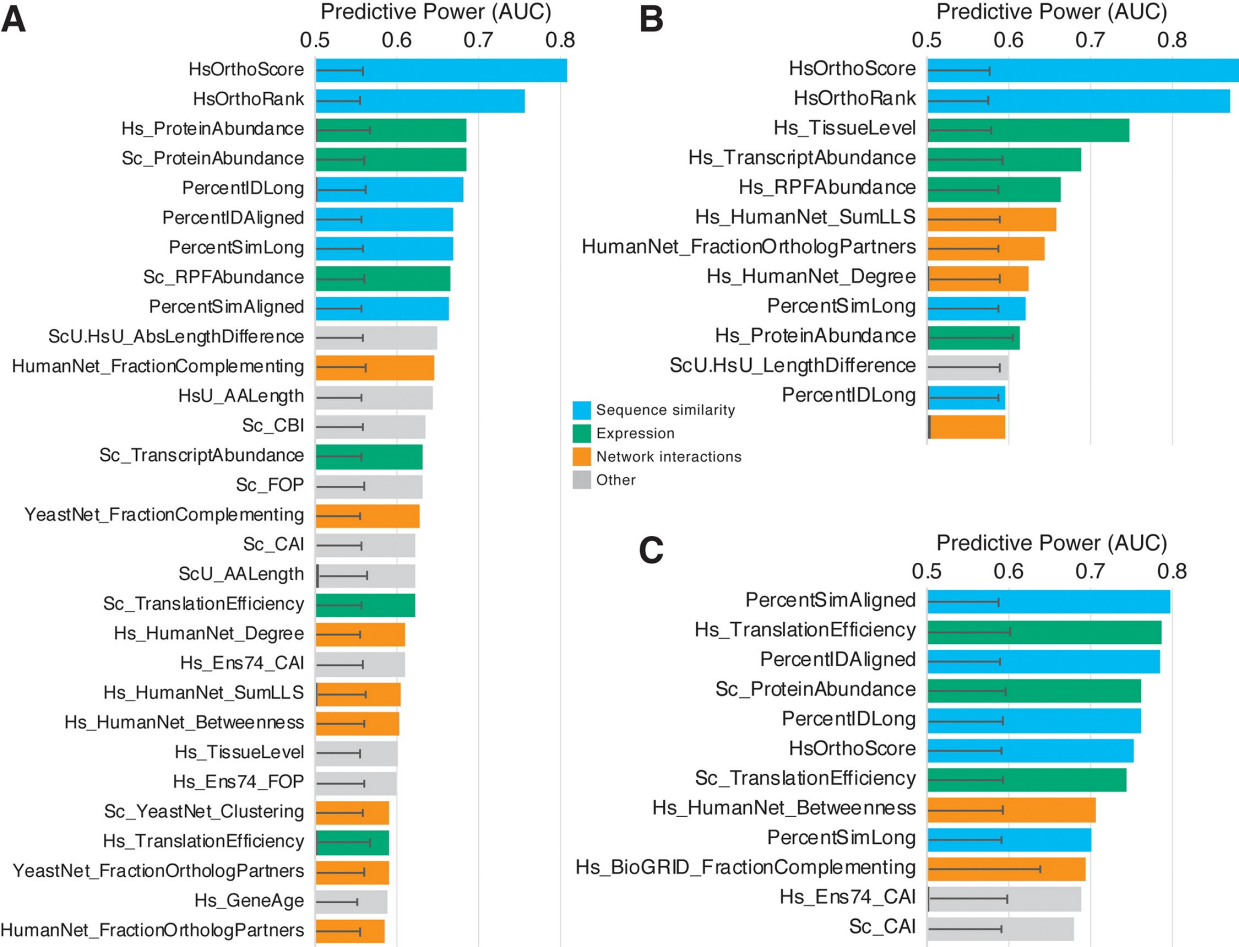
Replaceability of 1:2 orthologs is explained largely by relative divergence of human co-orthologs. (A) AUCs for the top 30 predictive features for median-collapsed 1:2 orthogroups are shown. The top two predictive features (HsOrthoScore and HsOrthoRank) for this class indicate that replaceability is driven largely by the nearness of the replacing human co-ortholog to the yeast gene relative to a nonreplacing co-ortholog (i.e., most 1:2 orthogroups have only one replacing human co-ortholog, and it is almost always the least-diverged one). (B) The observed trend is even more strongly demonstrated when analysis is restricted to the specific set of 1:2 orthogroups that display differential replaceability (i.e., one human co-ortholog replaces, and the other does not), with AUCs nearing 0.9. The extent of tissue-specific expression also becomes significantly predictive in this set, indicating that human co-orthologs that are more broadly expressed are more likely to replace than their more tissue-specifically expressed co-ortholog. The top 12 features are plotted. (C) More-diverged human co-orthologs in a 1:2 pair do replace in several cases (mostly those in which both co-orthologs replace). When restricting analysis to this set, it is apparent that the most predictive property is sequence similarity to the yeast ortholog, along with proteins that are translated efficiently and are typically in high abundance. Black overlapping bars indicate mean, and error bars indicate standard deviation for 1,000 shuffled AUC calculations for each feature. AUCs were calculated for *n* = 208 1:2 ortholog pairs, collapsed to 117 and 46 median metaorthologs ([A] and [B], respectively) depending on feature availability. AUCs for MDO assays in (C) were calculated for up to 104 MDO pairs (Materials and methods, **S3 Table**). The top 12 features are plotted. For full source data, see **S1 Data**. AA, amino acid; AUC, area under the receiver operating characteristic curve; CAI, codon adaptation index; CBI, codon bias index; Ens74, Ensembl genes version 74; FOP, frequency of optimal codons; Hs, *H*. *sapiens*; HsU, *H*. *sapiens* UniProt; LLS, log likelihood score; MDO, more-diverged ortholog; RPF, ribosome protected footprints; Sc, *S*. *cerevisiae*; ScU, *S*. *cerevisiae* UniProt.

**Fig 4:**
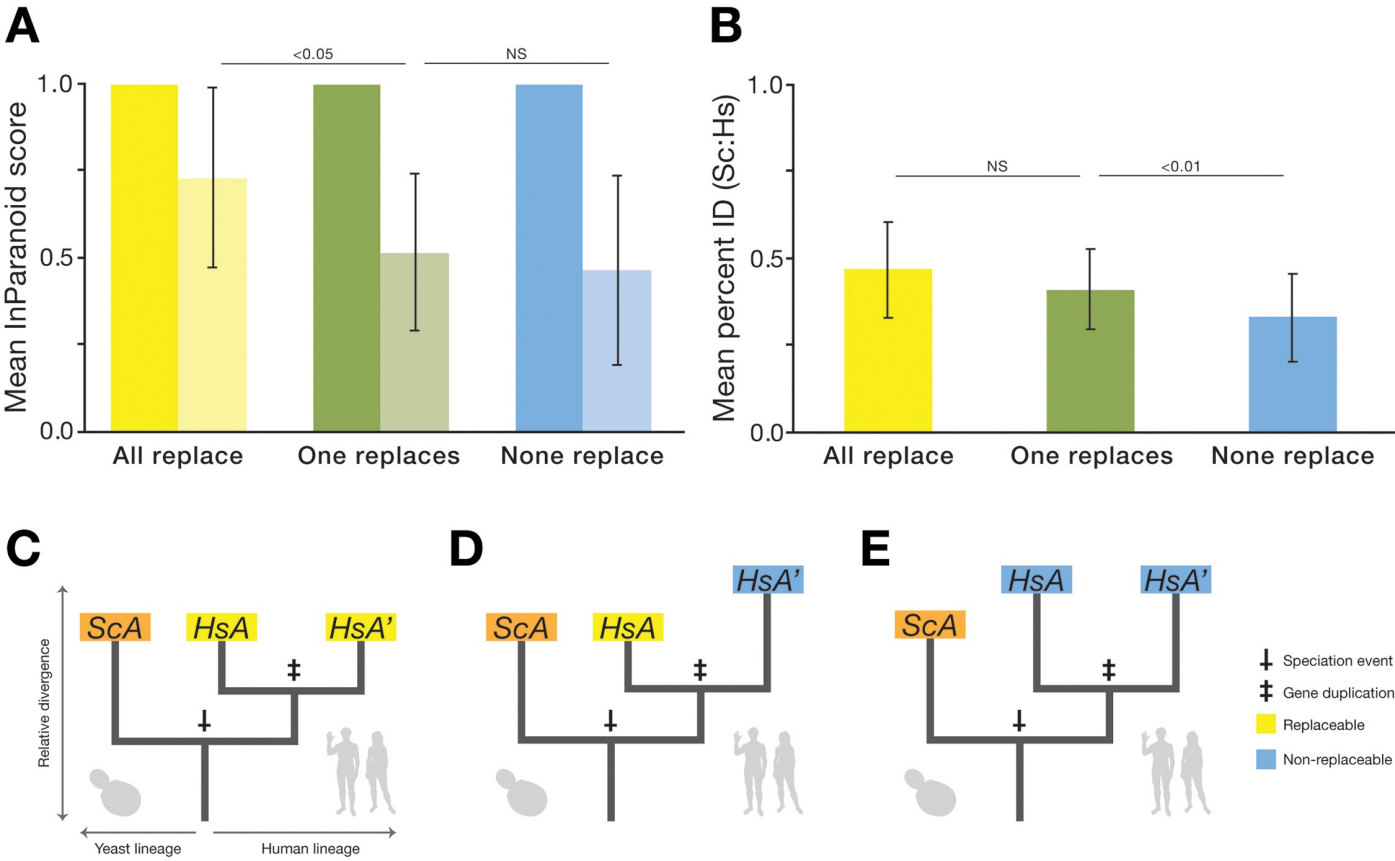
Replaceability is explained by relative divergence of 1:2 human co-orthologs from each other and their yeast ortholog. (A) The average ortholog score of the more-diverged (lighter color) 1:2 co-ortholog is more similar to the less-diverged co-ortholog for co-ortholog pairs that both replace than pairs in which only one or neither of the co-orthologs replace. (B) The average percent amino acid identity for 1:2 co-orthologs is not significantly different between the “all replace” (yellow) class and the “one replaces” (green) class, whereas the “none replace” (blue) class is slightly, but significantly, lower than either. Error bars indicate standard deviation. Raw data for (A) and (B) are available in **S2 Data**. (C–E) Phylogenetic models depicting gene trees for generic 1:2 orthogroups in the various replaceability classes. In (C), the human co-orthologs belong to the “both replace” class and are thus less diverged from each other but are on average similarly diverged from the yeast ortholog as the “one replaces” class (D), in which the co-ortholog more closely related to the yeast gene is the one that replaces. In (E), the “none-replace” co-orthologs are on average more diverged from the yeast gene. Hs, *H*. *sapiens*; HsA, *H*. *sapiens* co-ortholog A; HsA′, *H*. *sapiens* co-ortholog A′; NS, not significant; ScA, *S*. *cerevisiae* ortholog A.

Applying our computational analysis pipeline to the 1:2 orthogroups revealed several explanatory features. Most prominent in its explanatory ability, and in opposition to results from assays of 1:1 orthologs [5], was the relative divergence of the human co-orthologs from each other and from the yeast ortholog **(Fig 3A)**. In particular, the highest predictive power (measured as area under the ROC curve [AUC]) was seen for the InParanoid ortholog rank (Hs_OrthoRank), which ranks the human co-orthologs from more-diverged ortholog (MDO) to least diverged ortholog (LDO) from the yeast counterpart, and ortholog score (Hs_OrthoScore, a score ranging from 0 to 1, with scores closer to 1 meaning nearer to the yeast ortholog) **(Fig 3A)**. Of the 77 completely tested 1:2 orthogroups, there were 23 groups in which the two human genes displayed differential ability to replace (i.e., one human gene replaced, whereas the other did not). These groups are particularly interesting because they provide an opportunity to investigate properties that distinguish co-orthologs that can replace the yeast gene or not within the same orthogroup. To assess this difference, we performed the ROC analysis for each feature specifically on the set of differentially replaceable 1:2 orthogroups **(Fig 3B)**. In this restricted set, the relative co-ortholog divergence strengthened its standing as the most predictive feature in these orthogroups, demonstrating that the least diverged of two human co-orthologs was considerably more likely to replace **(Fig 3B)**. Indeed, in this differentially replaceable set, 86% of the replaceable human genes are also the LDO in their respective orthogroups (a trend similar to that observed by Hamza and colleagues [6]). Further, when we compared the divergence of the differentially replaceable set (“one replaces”) with the “both replace” and “none replace” sets, the average InParanoid ortholog scores of the MDO were similarly low for “one replaces” and”none replace” (0.52 and 0.47, respectively), whereas the MDO in the “both replace” class is notably more similar to the LDO (0.73) **(Fig 4A)**. The mean sequence identities between human and yeast orthologs for the “both replace” and “one replaces” classes are not significantly different, whereas the “none replace” class has a modestly lower mean identity to the yeast ortholog **(Fig 4B)**. Thus, although human co-orthologs in the three complementation classes have diverged from their yeast ortholog to a similar extent, those in the “both replace” class have maintained similarity to each other and are thus more likely to both replace **(Fig 4C–4E)**.

An additional significantly predictive feature appearing in the differentially replaceable 1:2 class pertains to the tissue-specific expression of the human orthologs. Specifically, co-orthologs with more widespread (less–tissue-specific) expression were more likely to replace **(Fig 3B)**. This feature is particularly predictive of the differentially replaceable set but was not a strong feature for the full 1:2 orthogroup results **(Fig 3A)**.

### More diverged yet replaceable 1:2 orthologs are highly expressed and more similar to their yeast counterparts

Despite the fact that in the majority of the 1:2 cases the least-diverged human co-ortholog replaced the corresponding yeast gene, there were several cases wherein the more-diverged co-ortholog was replaceable. Of 80 informative assays for human MDOs, 12 replaced the yeast ortholog. To determine what drives these replacements, we applied our ROC analysis scheme to the set, not restricting it to completely assayed groups or those in which the LDO also replaced. For these genes, the ability to complement is dominated by protein sequence similarity to the yeast ortholog as well as protein abundance features **(Fig 3C)**. These results are in line with the previous observation that human co-orthologs that both replace are more similar than those in which only one or none replaces, but they also suggest that these highly diverged co-orthologs retain high expression levels. Thus, some highly diverged human co-orthologs still seem to maintain ancestral functionality, irrespective of the other co-ortholog’s function.

### Human co-ortholog replaceability in 1:>2 orthogroups is marked by conserved interactions and subcellular localization

In the case of 1:>2 orthogroups, 137/170 human genes were successfully assayed, 30 of which replaced the yeast ortholog, whereas 107 did not (22% replaceable). There were 23 1:>2 groups completely assayed for all human co-orthologs, represented by 78 pairwise tests **(S5A and S4B Figs, S1 Table)**. From the yeast perspective, 18 of 36 yeast genes with one or more successful assays were replaceable by at least one human co-ortholog. Six of those were replaceable by only one co-ortholog, and only two yeast genes in the 1:>2 set were replaceable by all of their human co-orthologs **(S1 Table)**.

**Fig 5:**
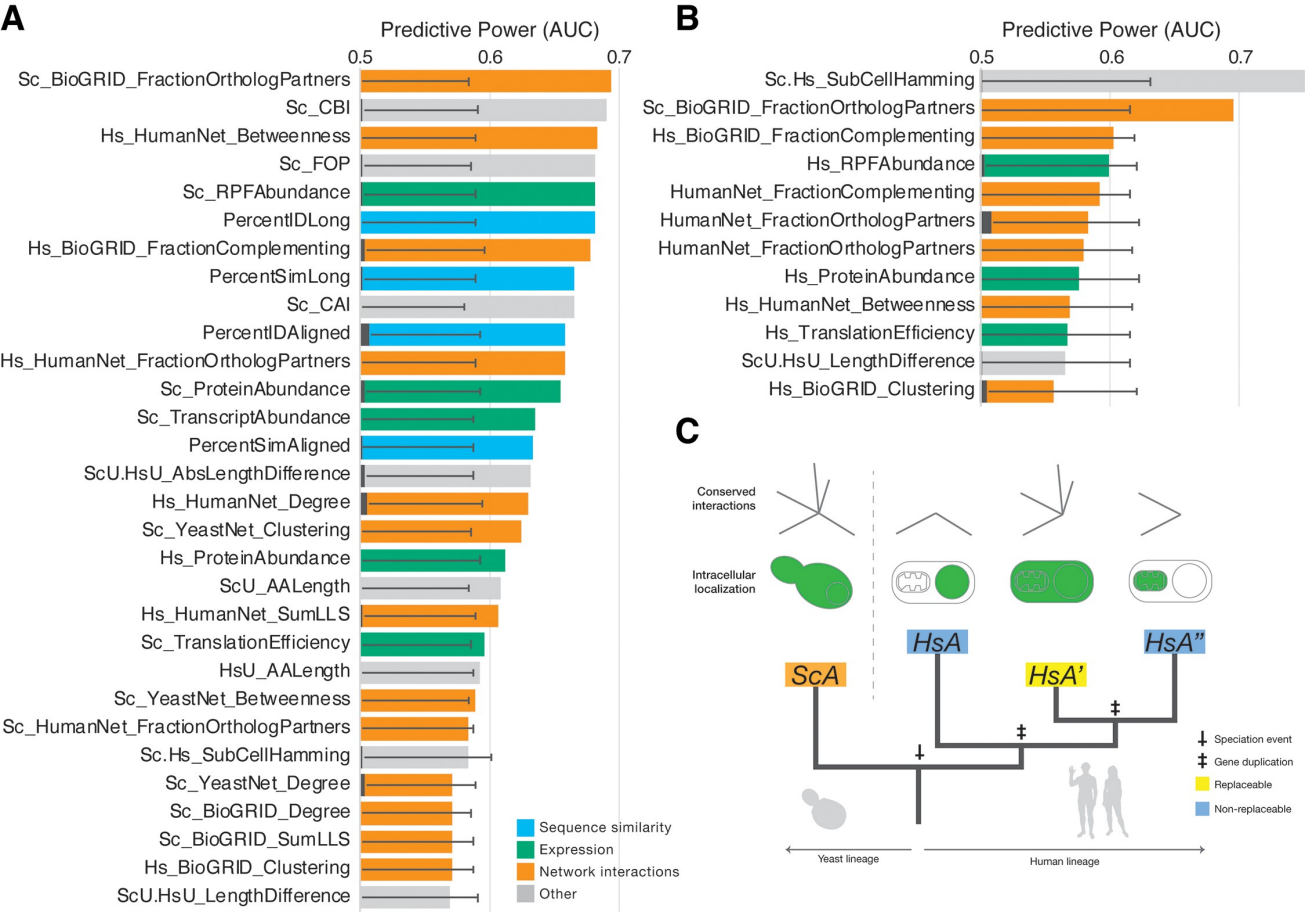
Replaceable 1:>2 human co-orthologs retain orthologous interaction partners and are more central in interaction networks. (A) AUCs of the top 30 predictive features for median-collapsed informative 1:>2 co-ortholog pairs. The top two most significant features demonstrate the importance of network context in retaining ancestral functions. Specifically, human co-orthologs in highly expanded gene families that have retained a higher fraction of orthologous protein interaction partners with their yeast ortholog (FractionOrthologPartners) are more likely to replace, as well as those that maintain higher centrality in functional interaction networks (betweenness). (B) Similar to the 1:2 case, we further restricted our analysis to a subset of median-collapsed 1:>2 orthogroups that had both replaceable and nonreplaceable human co-orthologs. In this set, the subcellular localization of the human proteins appears to be predictive, in that more broadly localized co-orthologs are more likely to replace than their more organellar-specific co-orthologs. Although this AUC is not significant (indicated by being just less than 2 standard deviations off the mean), it is an obvious trend and does overtake fraction of orthologous partners as the highest performing AUC for this set. (C) Phylogenetic model of a generic 1:M orthogroup showing that the replaceable human co-ortholog has retained more orthologous interactions in a network and is localized in a similar manner to the yeast ortholog. For AUC bar plots, black overlapping bars indicate mean, and error bars indicate standard deviation for 1,000 shuffled AUC calculations for each feature. AUCs were calculated for *n* = 170 1:>2 ortholog pairs, collapsed to 49 and 26 median metaorthologs ([A] and [B], respectively) depending on feature availability (Materials and methods, S3 Table). The top 12 features are plotted in (B). For full source data, see S1 Data. 1:M, one-to-many; AA, amino acid; AUC, area under the receiver operating characteristic curve; CAI, codon adaptation index; CBI, codon bias index; Ens74, Ensembl genes version 74; FOP, frequency of optimal codons; Hs, *H*. *sapiens*; HsA, *H*. *sapiens* co-ortholog A; HsA′, *H*. *sapiens* co-ortholog A′; HsA′′, *H*. *sapiens* co-ortholog A′′; HsU, *H*. *sapiens* UniProt; LLS, log likelihood score; RPF, ribosome protected footprints; ScA, *S*. *cerevisiae* ortholog A; ScU, *S*. *cerevisiae* UniProt.

Because orthogroups with more than two human members had differing rates of replaceability within them and not all human genes could be assayed, we again collapsed the orthogroups using median values of replaceable versus nonreplaceable genes in each 1:>2 orthogroup prior to analysis **(S6 Fig)**. Unlike for 1:2 orthogroups, but reminiscent of prior 1:1 findings, although sequence similarity appears near the most explanatory features, the ability of 1:>2 orthologs to replace was most strongly marked by functional context **(Fig 5A)**. In particular, the dominant predictive features included the fraction of conserved protein–protein interactions along with their centrality in their respective functional interaction networks and the number of interactions in those networks, albeit to a lesser extent. Specifically, human co-orthologs that have maintained a higher fraction of orthologous interaction partners relative to the yeast ortholog were more likely to replace. Further, human co-orthologs with relatively higher centrality in a functional network were more likely to be replaceable. Thus, in the case of highly expanded gene families, paralogs that maintain ancestral protein contacts and centrality in their interaction networks tend to be more replaceable **(Fig 5A)**, consistent with previous suggestions that “functional orthologs” retain a higher proportion of shared network contact [29] and observations that centrally placed proteins in interaction networks tend to carry out crucial cellular functions [30] that have been retained over vast evolutionary timescales.

We further sought to identify features that distinguished replaceable co-orthologs from nonreplaceable ones within the same orthogroup for the 1:>2 set. We again considered median features of both replaceable versus nonreplaceable co-orthologs within those 1:>2 orthogroups that showed differential replaceability in a manner similar to the differential 1:2 set. No features showed an AUC more than 2 standard deviations above the mean of permutation tests, likely due to the small size of this specific orthogroup set. Nonetheless, the strongest trend observed was for replaceable co-orthologs to be localized in more-similar subcellular compartments [31] in human cells as the yeast ortholog(s) **(Fig 5B)**. Our observations are consistent with a model that at least one co-ortholog in an expanded human gene family will tend to retain essential ancestral functionality by maintaining ancestral interactions and network centrality, as well as similar cellular localization **(Fig 5C)**.

### Simulations suggest most diverged duplicates are less likely to bind their ancestral interaction partners

**Fig 6:**
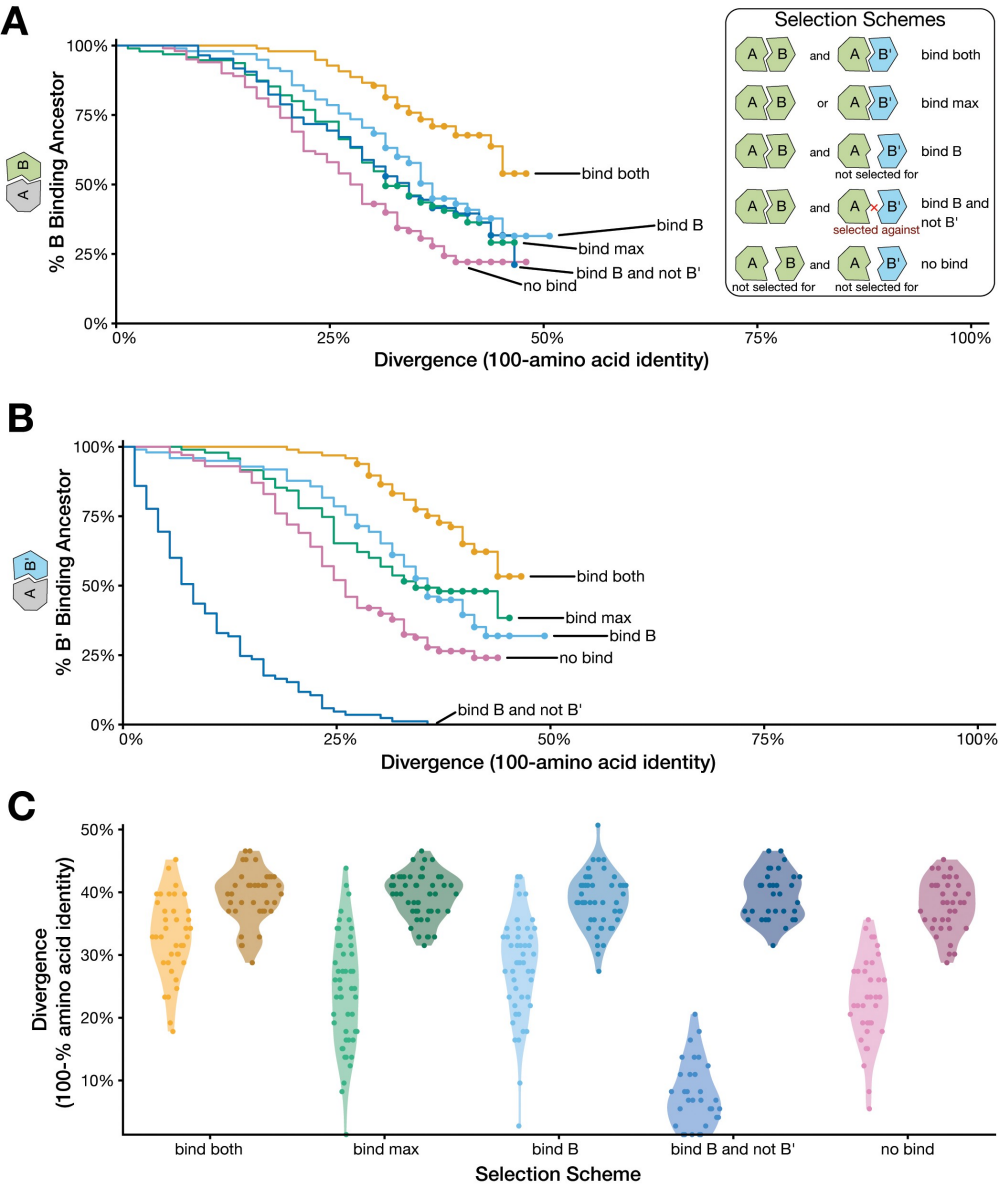
Simulated protein evolution suggests that diverged duplicates are less likely to bind their ancestral interaction partner. (A) Inset: Five different types of selection scenarios were considered for a heterodimeric protein complex, considering the effects of amino acid substitutions using the Rosetta molecular modeling platform. (1) AB and AB′ (bind both). (2) AB or AB′, and only the most stable binding interface is considered (bind max). (3) AB, but AB′ was not enforced (bind B). (4) AB, and AB′ is selected against (bind B and not B′). (5) Neither AB nor AB′ is selected for (no bind). Percent of simulations in which an evolved B subunit has the ability to bind the ancestor to A. Divergence is measured by the amount B has diverged from the ancestor of B. (B) Percent of simulations in which an evolved B′ subunit has the ability to bind the ancestor to A. Divergence is measured by the amount B′ has diverged from the ancestor of B. (C) Percent divergence of duplicates when only B or B′ is able to bind the ancestor A. Lighter hues denote a duplicate that is able to bind the ancestor of A, and darker hues denote the nonbinding duplicate. Data and scripts for these figures are available at the following link: https://github.com/a-teufel/Laurent_etal_2020. *Figure adapted from [35]*.

To assess whether these trends would be evident in a controlled evolution experiment, we performed in silico simulations of functional divergence in a duplicated gene family. We analyzed a small heterodimeric protein complex consisting of A and B subunits (the Ubiquitin-like protein, suppressor of Mif Two 3 [SMT3]–ubiquitin conjugating 9 [UBC9] protein complex [5, 32]) in which the subunit B was duplicated in silico to yield A, B, and B′. Using binding of A to B and/or B′ as a proxy for functionality, we carried out evolutionary simulations of molecular structural divergence (considering all atom models using the Rosetta molecular modeling suite [33, 34]). As described in [35], we examined functional replaceability across five different selection scenarios. All of the simulations assumed that selection acts on the stability of each subunit but differed in how they imposed selective pressure on binding. We ran 100 replicates for each of the five selection schemes and quantified replaceability of each of the subunits (measured as continued ability to bind to the ancestral partner) over sequence divergence **(Fig 6A and 6B).** Notably, selection for A to bind both B and B′ results in a continued ability for either B or B′ to bind the ancestral variant A, whereas application of diversifying selection to prevent binding to B′ results in a rapid decay in the ability of B′ to bind A **(Fig 6B)**.

We then looked across our simulated lineages at those cases in which one of the duplicates functionally replaces (i.e., binds to the ancestor) and the other does not. We found a systematic pattern across all five selection schemes that the nonbinding duplicate tends to be the more diverged one **(Fig 6C**; all pairwise comparisons within selection schemes are significant, *p* < 0.001 paired *t* test**)**. These results mirror our experimental humanization findings for the relative divergence of 1:2 human co-orthologs that the replaceable duplicate tends to be less diverged than the nonreplaceable duplicate **(Fig 4)**.

### Conclusions

By extending the scope of our systematic yeast humanization assays to include those yeast genes that have more than one human ortholog, we successfully added 337 human genes to our tested set **(Fig 2)**. We have therefore greatly expanded the set of human genes that can now be functionally studied in the simplified unicellular eukaryotic context of budding yeast by adding 50 novel human genes to those that can successfully replace their yeast ortholog. Overall, we found that yeast genes with duplicated human orthologs can be replaced by at least one co-ortholog at a slightly lower rate (40%) than 1:1 orthologs [5]. Of those that could be replaced, the clear majority was replaceable only by one or two co-orthologs, rather than being broadly replaceable by many human genes in the same family. This pattern of replaceability between and within orthogroups suggests divergence among gene family members away from ancestral functions, at least to the degree that yeast complementation assays can probe this effect [14, 15].

After testing many properties of the gene families for their ability to explain replaceability, our analysis revealed divergent patterns across groups that have two (1:2) or more than two (1:>2) human co-ortholog members. In the case of the 1:2 class, the top predictors were dominated by features that capture divergence from the yeast ortholog; in particular, we observed that the less-diverged variant strongly tended to be the replaceable one. This observation was supported by computational simulations of protein divergence after duplication, in which the least diverged of the duplicates retained ancestral binding ability in most cases. In addition to sequence divergence, we also observed a strong trend of replaceable 1:2 human co-orthologs to have broader, less–tissue-specific expression. Somewhat rarely, the more-diverged human co-ortholog could replace, and these tended to be more similar to their yeast counterpart and expressed at a higher level, perhaps due to retaining important ancestral functions. In the case of the 1:>2 set, replaceability was marked largely by network-based properties of the genes. Replaceable co-orthologs in this set seem to have retained more ancestral interaction partners as well as higher centrality, even across a billion years of divergent evolution, likely indicative of their functional importance. Although not significant by our criteria, we also observed replaceable 1:>2 co-orthologs being expressed in similar subcellular compartments to their corresponding yeast gene, highlighting a trend to maintain their ancestral localization.

Overall, our extended set of humanization assays and their analysis reveals trends in functional divergence among co-orthologs. We observed a strong tendency for orthogroups to exhibit only one or a few swappable human genes rather than many. We also extended our observation that network centrality and interaction properties aid in determining how ancestral gene function across orthologs is retained over deep evolutionary timescales. Our study was limited to those yeast genes that are known to be essential for growth, providing us with a straightforward phenotypic readout for our systematic screening strategy. Future studies incorporating custom phenotyping will be needed to determine whether similar rates and trends of replaceability are obeyed by nonessential genes.

Such assays help to advance our understanding of duplicate gene evolution in the billion years since yeast and humans diverged [36] and add powerful reagents to study myriad human processes and develop therapies in a simpler eukaryotic surrogate.

## Materials and methods

### Identifying orthologs

Orthologs were calculated with a local installation of InParanoid [1], using UniProt proteomes for the two species (downloaded November 2014). InParanoid identifies orthogroups between two species by first performing an all-versus-all BLAST search between the two species to identify bidirectional best hits (BBH). Each proteome is then subjected to an all-versus-all BLAST against itself to identify within-species homologs. The BBH pairs are used as seed matches, and any within-species genes from the self-BLAST that are at least as close to the gene of interest as its BBH in the other species are added to the ortholog group and termed an “in-paralog” or co-ortholog. We used the “table” output of InParanoid to identify orthogroup classes as follows: those groups with one listed yeast gene and two human genes were dubbed one-to-two (1:2) orthologs, whereas those with one yeast gene and more than two human genes were identified as one-to-more-than-two (1:>2) orthologs. Together, these two sets make up the 1:M ortholog class.

### ORFeome cloning

Human genes were obtained from the human ORFeome collection [23]. The ORFeome comprises a collection of *Escherichia coli* strains, each harboring a plasmid encoding a single human gene in a Gateway “entry” vector. Sequences cloned in the entry vectors are flanked by attL sites. To create expression vectors, each human entry vector was isolated from *E*. *coli*, added to a Gateway LR reaction with a Gateway “destination” vector, and transformed into competent *E*. *coli* to obtain expression clones [37]. As ORFeome clones lack stop codons, we modified the Advanced Yeast Gateway kit [25] pAG416-GPD-ccdB destination vector, which does not encode a stop codon immediately outside of the cloning region, resulting in a tail of approximately 60 amino acids being added to any protein expressed from it. We thus mutagenized the vector downstream of the cloning region to introduce a stop codon, shortening the tail to six amino acids (this plasmid is termed pAG416-GPD-ccdB+6Stop) [5]. Entry and expression clones were verified by sequencing into the gene sequence from the upstream and downstream regions of the plasmid.

### MGC cloning

For human genes not available in the ORFeome, we obtained clones from the Mammalian Gene Collection [24], a collection of sequence-verified human cDNA sequences. To obtain entry vectors for these genes, we designed primers for each gene that would amplify the coding sequence while adding attB sites to either end of the human gene and performed PCR using the MGC plasmid as a template. PCR products were combined with plasmid pDONR221 in a Gateway BP reaction [37] and transformed to *E*. *coli* to obtain entry clones. The entry clones were then combined in a Gateway LR reaction with p416-GPD-ccdB+6Stop and transformed to *E*. *coli* to obtain expression clones. Each entry and expression clone was verified by sequencing the clone boundaries at each end of the gene.

### Functional replaceability assays

Yeast strains were grown in a 96-well format in YPD medium supplemented with G418 (200 μg/ml). The strains were transformed with matched expression clones or empty control vectors and selected on minimal medium lacking uracil. Complementation assays were performed as follows:

#### TS assays

Each of the strains in the TS strain collection [27] encode a yeast protein with a mutation that allows growth at permissive temperatures of 22–26°C but not at the restrictive temperature of 35–37°C. We therefore identified human genes capable of rescuing growth of the mutant at restrictive temperature on selective plates. Each strain was transformed and assayed separately with either the human gene-expressing vector or the empty vector control by growing transformed strains in the following manner: −Ura dextrose medium at the permissive temperature (26°C), which serves as a control for transformation efficiency and/or toxicity because both the yeast and the human gene are expressed; and −Ura dextrose medium at the restrictive temperature (37°C), testing for human gene functional replacement under conditions in which the corresponding yeast gene is nonfunctional.

#### hetKO assays

Strains in the hetKO collection (obtained from ATCC) are heterozygous diploid strains, each harboring one allele of a yeast gene knocked out by replacement with the KanMX kanamycin-resistance cassette, allowing for selection on G418 [26]. We transformed human expression clones or an empty control vector into appropriate strains and selected on −Ura G418 medium in a 96-well format. Transformants were then replated on GNA-rich presporulation medium containing G418 and 50 mg/L histidine. Individual colonies were then inoculated in liquid sporulation medium containing 0.1% potassium acetate, 0.005% zinc acetate, and incubated with vigorous shaking at 26°C for 3–5 days, after which sporulation efficiency was estimated by microscopy, and the mixture was then resuspended in water and equally plated on two assay conditions: (1) −G418 Magic Marker dextrose medium (−His −Arg −Leu +Can −Ura) incubated at 30°C. The haploid spores that carry the wild-type allele grow in this medium, providing us with the control for sporulation efficiency. This condition also assays for toxicity of the human gene if the haploid spores fail to grow. (2) +G418 Magic Marker dextrose medium (−His −Arg −Leu +Can −Ura) containing 200 μg/ml G418. In the absence of the human gene (as for control transformants), the resulting haploid knockout strain is expected not to grow, providing an assay for replaceability in strains expressing the human gene. Cases with approximately equal numbers of colonies growing in the absence or presence of G418 were considered functional replacements. For cases with ambiguous growth (marked by moderate numbers of isolated colonies growing on the +G418 medium relative to −G418 medium), we screened varying quantities of the sporulation mixtures.

Positive assays were verified independently. Individual colonies were isolated from selective plates and were assayed for growth defects on YPD or Magic Marker medium + G418 (**Fig 1B, S4 and S5 Figs**). After growth on YPD + G418, each strain was spotted on 5-FOA agar to test plasmid dependency.

### Tetrad dissection and plasmid loss assays

For human gene replaceability assays performed in the yeast hetKO collection that were ambiguous in our large-scale screen, we performed tetrad dissections to more clearly test for complementation (**S2 Fig**). In total, 33 human genes were assayed and analyzed. We transformed each human expression clone or empty vector control into the appropriate yeast strains and selected on Synthetic Complete (SC)-Ura + G418 (200 μg/ml) to select for the human gene expression vector (CEN, Ura+) and yeast gene knockout (KanMX marker) simultaneously. Transformants were then plated on GNA-rich presporulation medium containing G418 (200 μg/ml). Individually isolated colonies were inoculated into liquid sporulation medium containing 0.1% potassium acetate, 0.005% zinc acetate, and were incubated with vigorous shaking at 25°C for 3–5 days. Following this, sporulation efficiency was estimated by microscopy, and successful sporulations were subjected to tetrad analysis. In all, 15–20 μL of each sporulation was digested with an equal volume of Zymolyase (5 mg/ml stock) for 30–45 minutes to remove the ascus coats. The digestions were diluted 1:1 with sterile water, after which 20–30 μL of the Zymolyase-treated spore mix was carefully applied to a tilted YPD plate using a pipette, allowing the droplet of cell suspension to gently run down the agar surface. The plates were dried and visualized on the tetrad dissection microscope. For each human gene, a minimum of five tetrads were dissected. Dissections were selected and replica plated both on 5-FOA (for plasmid counterselection) and YPD + G418 (for yeast null allele selection). A successful complementation consists of 2:2 segregation with survival on YPD + G418 and failure to grow on 5-FOA **(S2 Fig, S1 Table)**. We subsequently performed quantitative growth assays (in triplicate) on tetrads passing the 5-FOA/G418 segregation test. Each humanized tetrad was grown in three different media conditions: YPD, YPD + G418, and SC-Ura. Subsequently, dissections were replica plated to SC-URA to orthogonally confirm plasmid dependencies in the G418-resistant clones. Each medium-specific growth profile (shown in one of the conditions as in **S4 and S5 Figs**) was analyzed and quantified to detect any growth defects in yeast. In all, 14 out of 33 human genes assayed in this manner showed functional replaceability of the yeast gene function **(S1 Table)**.

### Isolating haploid humanized yeast strains and quantitative growth curve assays

Select yeast hetKO strains carrying human gene expression vectors (CEN, Ura+) showing functional replaceability were sporulated and plated on independent petri plates to obtain single colonies. Each colony was tested for plasmid loss in the presence of 5-FOA. Colonies that did not grow in the presence of 5-FOA were subjected to further analysis to quantitatively measure their growth rates. Yeast strains were either precultured in liquid YPD + G418 (200μg/ml) or −Ura dextrose selective medium + G418 (200 μg/ml) for 2 hours or overnight, respectively. Each culture was diluted in YPD or −Ura dextrose medium to an OD of approximately 0.1 in 100 or 150 μL total volume in a 96-well plate. Plates were incubated in a Synergy H1 shaking incubating spectrophotometer (BioTek), measuring the OD every 15 minutes over 48 hours. Growth curves were performed in triplicate for each strain by splitting the preculture into three independent cultures for each 48- to 60-hour time course (**S4A, S5A and S5C Figs**).

In the case of TS humanized yeast strains, the growth assays were performed first at permissive temperatures (25–26°C) for a 48- to 60-hour time course. These growth assays were largely identical to the empty vector transformed yeast strains. The strains were then shifted to a restrictive temperature of 37°C, and we similarly repeated the growth assay for a 48- to 60-hour time course (**S4B and S5B Figs**). Growth curves were performed in triplicate for each strain by inoculating cells from the same preculture into three independent cultures.

For computational analyses of the trends underlying replaceability, we computed a diverse set of features **(S3 Table)**, based in part on the previously published work [5], as follows:

### Sequence properties

Sequence features for human genes obtained from the human ORFeome [23] were calculated using the ORFeome-provided fasta file for the sequences, which was downloaded from http://horfdb.dfci.harvard.edu/hv7/docs/human_orfeome71.tar.gz. For clones not obtained from the human ORFeome collection, we calculated sequence features using the longest annotated transcript or its translation from Ensembl version 74, available at http://Dec2013.archive.ensembl.org/index.html. To analyze protein features, we translated the above nucleotide sequence for each gene using the standard genetic code. The following protein sequence features were considered:

Sequence length:

Sc_Length
Hs_Length
Sc-Hs_LengthDifference
Sc-Hs_AbsLengthDifference

Length was calculated as the count of amino acids in the protein. LengthDifference was calculated as the length of the human ortholog protein subtracted from the length of the yeast ortholog. AbsLengthDifference is the absolute value of LengthDifference.

Sequence similarity:

Sc-Hs_PercentIDLongest
Sc-Hs_PercentIDAligned
Sc-Hs_PercentSimLongest
Sc-Hs_PercentSimAligned
Hs_Orthoscore
Hs_OrthoRank

Orthologous pairs were first identified by InParanoid [1]. Global alignments for all preidentified ortholog pairs were then calculated using NWalign (http://zhanglab.ccmb.med.umich.edu/NW-align/) with BLOSUM62 and gap open penalty of −11 and extension −1. Identity and similarity were calculated as the fraction of identical amino acids and amino acids with positive BLOSUM score in the alignment, respectively. “Longest” refers to amino acid identity or similarity calculated as a fraction of the longer of the two orthologs. “Aligned” refers to calculating identity or similarity calculated as a fraction of the length of the aligned region of the sequences. OrthoScore and OrthoRank refer to the scores assigned by InParanoid. For each orthogroup, InParanoid calculates a confidence score between 0 and 1 for each in-paralog that represents how similar it is to the seed ortholog. Rank is simply a ranked ordering of the in-paralogs of a group based on their orthoscore, with 1 being the LDO, and higher values being further away from the seed.

Codon usage:

Sc_CAI
Sc_CBI
Sc_FOP
Hs_CAI
Hs_CBI
Hs_FOP

The above listed properties were calculated from the amino acid sequences using CodonW (https://sourceforge.net/projects/codonw/). Properties for the human genes were calculated using the yeast codon optimality table as a measure of closeness or divergence from yeast optimality.

### Network properties

Network features were calculated using custom Python scripts, typically utilizing the package “networkx” (available from https://github.com/networkx/documentation/tree/gh-pages/latest/_downloads). When applicable (e.g., HumanNet, YeastNet), provided weights of interactions were taken into account when calculating these features. Otherwise, a default weight of 1.0 was used. Network features were defined as follows: Degree represents the count of interaction partners for a node in a given network. Betweenness represents network centrality, a measure of how central in a network a given node is, calculated as the number of shortest paths between all node pairs in a network that pass through a given node. Clustering represents the node clustering coefficient, calculated as the fraction of edges that could possibly be present in a node’s neighborhood that are actually present. FractionComplementing is the fraction of interaction partners observed to complement (including results obtained in our 1:1 humanization assays [5]). FractionOrthologPartners is the fraction of a gene’s interaction partners that have orthologs in the other species and interact with the gene of interests’ ortholog in the corresponding network (i.e., if gene Sc-*A* interacts with Sc-*X*, Sc-*Y*, and Sc-*Z*, and Hs-*A* interacts with Hs-*X* and Hs-*Y* but not Hs-*Z* [which is a legitimate gene], the FractionOrthologPartners for Sc-*A* is 2/3 or 0.66). It represents a measure of the degree to which interactions are maintained between orthologs in the two species.

BIOGRID:

[Hs|Sc]_BIOGRID_[Degree|Betweenness|Clustering|FractionComplementing|FractionOrthologPartners]

The above listed properties were calculated from interactions present in BIOGRID 3.1.93 [38], using only those interactions annotated as “physical interactions.”

Functional networks:

[Hs|Sc]_*Net_[Degree|Betweenness|Clustering|FractionComplementing|FractionOrthologPartners]

Human and yeast functional gene network features were calculated based on HumanNet [39] and YeastNet [40], respectively. The final sum log-likelihood score reported for each interaction was employed as an edge weight for calculations.

Abundance properties:

[Hs|Sc]_ProteinAbundance
[Hs|Sc]_TranscriptAbundance
[Hs|Sc]_RPFAbundance
[Hs|Sc]_TranslationEfficiency
ScHs_SubCellHamming

Protein abundances were used as reported by Kulak and colleagues [41]. TranscriptAbundance, Ribosome Protected Fragments (RPF), Abundance, and TranslationEfficiency were calculated from Guo and colleagues (human) [42] and Ingolia and colleagues (yeast) [43]. Translation efficiency was calculated as the ratio of RPF reads to mRNA reads. SubCellHamming is a measure of the difference in subcellular localization between a yeast–human ortholog pair, calculated using data from the COMPARTMENTS database ([31], downloaded in February 2017). We utilized the “benchmark” sets to create, for each protein in its respective species, a binary vector of subcellular localization across 11 compartments (cytoskeleton, cytosol, endoplasmic reticulum, extracellular space, Golgi apparatus, lysosome, mitochondrion, nucleus, peroxisome, plasma membrane) that were common to both human and yeast in the database. Then, for each ortholog pair, we calculated the hamming distance between their respective subcellular localization vectors as the SubCellHamming value.

### Calculating predictive strength of features

The predictive power of each feature was calculated as the AUC when treating that feature as an individual classifier. Each feature was sorted in both ascending and descending directions, retaining the direction providing an AUC > 0.5. To assess significance, a permutation procedure was performed as follows: for each feature, the replaceable/nonreplaceable status of each ortholog pair was shuffled (retaining the original ratio of replaceable-to-nonreplaceable assignments), and the AUC was calculated. The shuffling procedure was carried out 1,000 times for each feature, and the mean AUC values and their standard deviations were reported.

#### Features of expanded orthogroups

In order to avoid overweighting expanded gene families and compensate for uneven sampling, we considered median properties of genes in each orthogroup as follows.

For each orthogroup, we collapsed all yeast–human ortholog pairs with the same status (replaceable or not) into a single case, with the value of each feature calculated as the median value of the collapsed ortholog pairs for that feature from the original table. Thus, each 1:M orthogroup is collapsed to be represented by either two pairs (complement and noncomplement) or one pair (either complement or noncomplement).

### Simulations

We constructed a simulation of protein evolution in which one member of an interacting pair was duplicated. The simulation was initialized with the yeast SMT3–UBC9 complex, a small heterodimeric protein complex, as the resident genotype (PDB: 2EKE) [32]. This complex initially had two subunits, which we refer to as A and B. We duplicated the B subunit and refer to it as B′. Our simulation protocol and setup [35] is based on an accelerated origin-fixation model [5, 44]. Here, we further analyzed five of the previously published sets of 100 simulation trajectories [35] (summarized in the **Fig 6A Selection Schemes panel**). Briefly, under the first selection scheme, we enforced selection for A to bind B and for A to bind B′ (bind both). In the second scheme, selection acts on the ability of A to bind B or A to bind B′, and the maximum stability of those interactions was considered (bind max). In the third scheme, the ability of A to bind B was selected for, but the ability of A to bind B′ was not (bind B). In the fourth scheme, the ability of A to bind B was also selected for, but the ability of A to bind B′ was selected against (bind B and not B′). We also implemented a control selection scheme in which selection does not act on the ability of A to bind either B or B′ (no bind). We performed 100 replicates of each of these selection schemes.

The percent of simulations in which an evolved duplicate has the ability to bind the ancestral partner **(Fig 6A and 6B)** corresponds to S7A and S7B Fig in [35]. We further analyzed these data by examining instances in which only one of the duplicates is able to bind the ancestral partner at the end of the simulation run. We recorded each duplicate’s divergence, defined as the fraction of amino acid positions that were nonidentical between the initial and final sequences of a simulation run **(Fig 6C)**. For each selection scheme, the distribution of the divergence of duplicates that can bind the ancestral partner was compared with the distribution of divergence of duplicates that cannot bind the ancestral partner with a paired *t* test.

Simulation data and scripts are available at https://github.com/a-teufel/Laurent_etal_2020.

## Supporting information

S1 Fig Count of orthogroups with the corresponding number of human gene members. The vast majority of one to multiple orthologs are of the 1:2 variety (1 yeast gene to 2 human co-orthologs), whereas the rest were classified as 1:>2 (1 yeast gene to >2 human co-orthologs). These two groups were considered separately during feature analysis.
(TIF)

S2 Fig Detailed overview of the ortholog groups in this study. (A) Venn diagram indicating the overlap of essential yeast genes with their human orthologs in various ortholog classes. Ortholog definitions are based on InParanoid [1], whereas essentiality of yeast genes is based on the systematic yeast knockout study by Winzeler and colleagues [22]. (B) A thorough breakdown of the numbers of ortholog pairs identified through to those resulting in informative assays.
(TIF)

S3 Fig Detailed illustration of yeast gene replaceability, tetrad dissection, and plasmid loss assays performed in a yeast hetKO collection. (A) Yeast strains were grown in 96-well plates in selective medium (YPD + 200 μg/ml G418). The matched orthologous human gene expression clones in 96-well plates were transformed into the appropriate yeast strains, followed by selection on the appropriate dropout medium (SD-Ura). The resulting transformants were spotted onto selective medium with appropriate markers to assay complementation. (B) In the case of the yeast hetKO collection, replacement was further verified by carrying out tetrad dissection followed by testing for plasmid dependency (selection on 5-FOA). Representative tetrad dissection assays are shown (right). Hs-*YIPF5* functionally replaces its yeast counterpart. Dissected tetrads showed 2:2 segregation, with tetrads 1 and 3 containing the wild-type yeast allele (surviving on 5-FOA), whereas tetrads 2 and 4 contain the KanMX allele (yeast null allele) complemented by the human gene (surviving on YPD + G418). 5-FOA was used for counterselecting the human gene expression vector (CEN6, Ura), whereas YPD + G418 was used to select for the yeast null allele (KanMX selection), respectively. However, in cases on noncomplementers (e.g., Hs-*CDC14A*), only two yeast spores (carrying the wild-type yeast allele) survive on YPD and fail to grow on YPD + G418 but grow on 5-FOA, indicating plasmid loss. (C) Each confirmed humanized haploid yeast strain was assayed for growth defects to quantify the replaceability. 5-FOA, 5-flouroorotic acid; *CDC14A*, human dual specificity protein phosphatase *CDC14A* gene; hetKO, heterozygous diploid knockout, Hs, *H*. *sapiens*; SD, synthetic defined; Ura, uracil; *YIPF5*, human *YIPF5* gene; YPD, yeast extract peptone dextrose.
(TIF)

S4 Fig Quantitative growth assays of all humanized yeast strains belonging to the 1:2 class. (A) Growth curves of humanized haploid yeast strains obtained after sporulation and tetrad dissection and followed by 5-FOA screening of yeast hetKO strains. The assays were performed in the presence of G418 (yeast gene–absent and human gene–present condition). Haploid yeast gene deletion strains carrying plasmids expressing functionally replacing human genes (colored red or purple solid lines) generally exhibit comparable growth rates to the wild-type parental yeast strain BY4741 (black dotted lines), except in the case of Hs-*ALAS2*, which shows reduced ability to replace the orthologous yeast gene compared with Hs-*ALAS1*. Mean and standard deviation plotted from triplicate assays. (B) Growth curves of humanized haploid temperature-sensitive yeast strains performed at a restrictive temperature of 37°C (yeast gene–inactive and human gene–present condition). Temperature-sensitive haploid yeast strains carrying plasmids expressing functionally replacing human genes (colored red, purple, or blue solid lines) generally exhibit comparable growth rates to the wild-type parental yeast strain BY4741 (black dotted lines). The yeast strains harboring empty vector without a corresponding human gene, showing no or poor growth in restrictive temperatures of 37°C (gray solid lines), serve as controls. In total, 208 individual assays were performed (as two independent biological replicates). In all, 45 human genes showed functional replaceability in either assay, whereas 127 did not, and 36 human gene complementation assays were noninformative (cases in which the control experiments did not behave appropriately). Raw data for growth curves are available in **S3 Data**. 5-FOA, 5-flouroorotic acid; *ALAS1*, human 5-aminolevulinate synthase *ALAS1* gene 1; *ALAS2*, human 5-aminolevulinate synthase *ALAS1* gene 2; hetKO, heterozygous diploid knockout; Hs, *H*. *sapiens*.
(TIF)

S5 Fig Quantitative growth assays of all humanized yeast strains belonging to 1:>2, M:M, and M:1 orthogroups. In total, 170 individual assays were performed (as two independent biological replicates): 32 human genes showed functional replaceability in either assay, whereas 105 did not, and 33 human gene replaceability assays were noninformative (cases in which the control experiments did not behave appropriately). (C) Growth curves of humanized haploid yeast strains (M:1 and M:M class) obtained after sporulation and/or tetrad dissection and followed by 5-FOA screening of hetKO strains. The assays were performed in the presence of G418 (yeast gene–absent and human gene–present condition). In total, 40 individual assays were performed (as two independent biological replicates). Three human genes showed functional replaceability, whereas 26 did not, and 11 human gene replaceability assays were noninformative (cases in which the control experiments did not behave appropriately). Mean and standard deviation plotted with *N* = 3. (A) Growth curves of humanized haploid yeast strains (1:>2 class) obtained after sporulation and/or tetrad dissection followed by 5-FOA screening of hetKO strains. The assays were performed in the presence of G418 (yeast gene–absent and human gene–present condition). Haploid yeast gene deletion strains carrying plasmids expressing functionally replacing human genes (colored red, purple, and blue solid lines) generally exhibit comparable growth rates to the wild-type parental yeast strain BY4741 (black dotted lines), except in the case of Hs-*PABPC1* and Hs-*PABPC3*, which shows reduced ability to replace the orthologous yeast gene. Mean and standard deviation plotted from triplicate assays. (B) Growth curves of humanized haploid temperature-sensitive yeast strains (1:M class) performed at a restrictive temperature of 37°C (yeast gene–inactive and human gene–present condition). Temperature-sensitive haploid yeast strains carrying plasmids expressing functionally replaced human genes (colored red, purple, or blue solid lines) generally exhibit growth rates comparable to their parental wild-type yeast strain BY4741 (black dotted lines), except in the cases of Hs-*CDK1*, Hs-*SEC11A*, and Hs-*SEC11C*, which showed reduced ability to functionally replace their orthologous yeast gene. Yeast strains harboring an empty vector without a corresponding human gene, showing no or poor growth at a restrictive temperature of 37°C (gray solid lines), serve as controls. (Raw data for growth curves are available in **S3 Data**.) 1:M, one-to-many; 5-FOA, 5-flouroorotic acid; *CDK1*, human cyclin-dependent kinase 1 gene; hetKO, heterozygous diploid knockout; Hs, *H*. *sapiens*; M:1, many-to-one; M:M, many-to-many; *PABPC3*, human polyadenylate-binding protein 3 gene; *SEC11A*, human signal peptidase complex catalytic subunit A gene; *SEC11C*, human signal peptidase complex catalytic subunit C gene.
(TIF)

S6 Fig Demonstration of the “median-collapse” feature table procedure. (A) An example subset of full ortholog data for two orthogroups in the 1:M ortholog class. (B) The same data as in (A) following median collapse. Orthogroup 218 has been collapsed to a single metaortholog pair with the status “noncomplement” because all human co-orthologs failed to complement. Group 249 has been collapsed to two metaortholog pairs because there were co-orthologs that complemented and did not. Metaortholog feature values are the medians of the full ortholog values with the same status. 1:M, one-to-many.
(TIF)

S1 Table Complementation results for all assays. We list the results of each assay background (TS or hetKO) as well as tetrad complementation reassay status and the final overall status used for subsequent analyses. Also listed is the literature status of genes found in YeastMine as previously reported literature assays. Those without a literature status are considered novel to this study. hetKO, heterozygous knockout; TS, temperature-sensitive.
(XLSX)

S2 Table Literature assay status from YeastMine. We list the results of 1:M ortholog assays previously reported in the literature as found via the YeastMine database. We also indicate whether these match our reported assays and the accuracy calculation reported in the text. 1:M, one-to-many.
(XLSX)

S3 Table Protein and ortholog properties. Includes all properties assembled and calculated for all proteins or ortholog pairs used for ROC analyses. ROC, receiver operating characteristic.
(XLSX)

S1 Data AUC tables for various ortholog classes. The various tabs in the file list the full AUC calculations for all features for the given ortholog classes as found in the main figures. AUC, area under the receiver operating characteristic curve.
(XLSX)

S2 Data Divergence data for 1:2 class comparison. This table corresponds to the raw data used to generate **Fig 4A and 4B**. The tabs are named according to which group of 1:2 orthologs they belong to.
(XLSX)

S3 Data Growth curve data for replaceable orthologs. These files contain the raw growth curve data used to generate **S4 and S5 Figs**.
(ZIP)

## Abbreviations

1:M: one-to-many
AUC: area under the receiver operating characteristic curve
CAI: codon adaptation index
CBI: codon bias index
CSTF2: Cleavage stimulating factor subunit 2
CSTF2T: Cleavage stimulating factor subunit 2 tau subunit
Ens74: Ensembl genes version 74
ERD2: ER lumen protein-retaining receptor, endoplasmic reticulum retention defective 2
FOP: frequency of optimal codons
GPD: glyceraldehyde-3-phosphate dehydrogenase
hetKO: heterozygous diploid knockout
Hs: *Homo sapiens*
HsA: *H*. *sapiens* co-ortholog A
HsA′: *H*. *sapiens* co-ortholog A′
HsU: *H*. *sapiens* UniProt
KDELR1: ER lumen protein-retaining receptor 1
KDELR2: ER lumen protein-retaining receptor 2
KDELR3: ER lumen protein-retaining receptor 3
LDO: least-diverged ortholog
LLS: log likelihood score
M:1: many-to-one
MAGE: melanoma antigen gene
MDO: more-diverged ortholog
M:M: many-to-many
PRE6: Proteasome subunit alpha type-4
PRO3: Delta 1-pyrroline-5-carboxylate reductase
PSMA7: Proteasome subunit alpha type-7
PSMA8: Proteasome subunit alpha type-8
PYCR1: Pyrroline-5-carboxylate reductase-1
PYCR2: Pyrroline-5-carboxylate reductase-2
ROC: receiver operating characteristic
Sc: *Saccharomyces cerevisiae*
ScA: *S*. *cerevisiae* ortholog A
ScU: *S*. *cerevisiae* UniProt
SMT3: Ubiquitin-like protein, suppressor of Mif Two 3
TS: temperature-sensitive
UBC9: ubiquitin conjugating 9
