# Supplementary figures and images for: Humanization of yeast genes with multiple human orthologs reveals functional divergence between paralogs
Source: PLoS Biol. 2020 May 18;18(5):e3000627. doi: 10.1371/journal.pbio.3000627 (PMC7259792; doi:10.1371/journal.pbio.3000627)

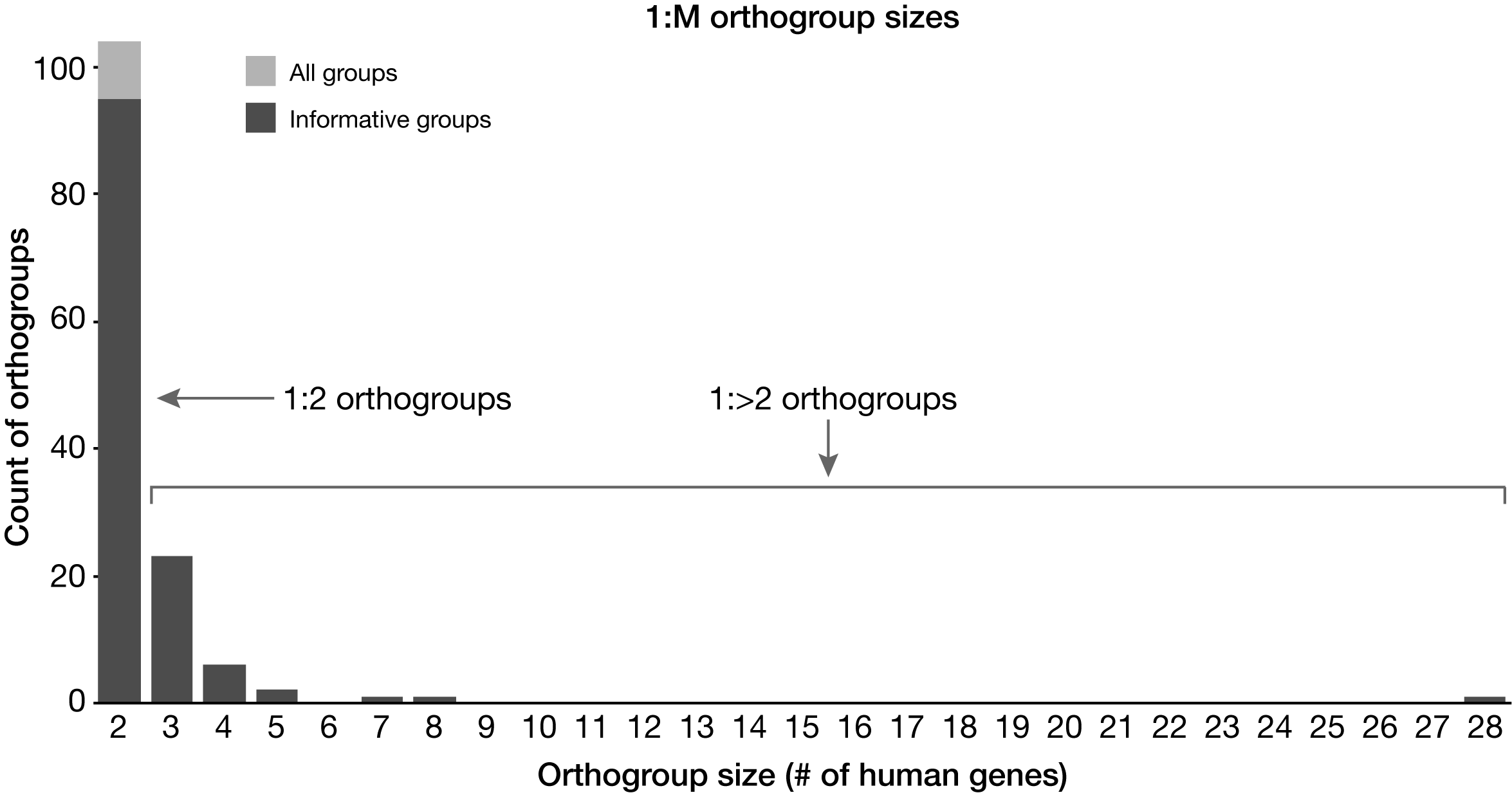

Supplement: S1 Fig — The vast majority of one to multiple orthologs are of the 1:2 variety (1 yeast gene to 2 human co-orthologs), whereas the rest were classified as 1:>2 (1 yeast gene to >2 human co-orthologs). These two groups were considered separately during feature analysis. (TIF) [file pbio.3000627.s001.tif]

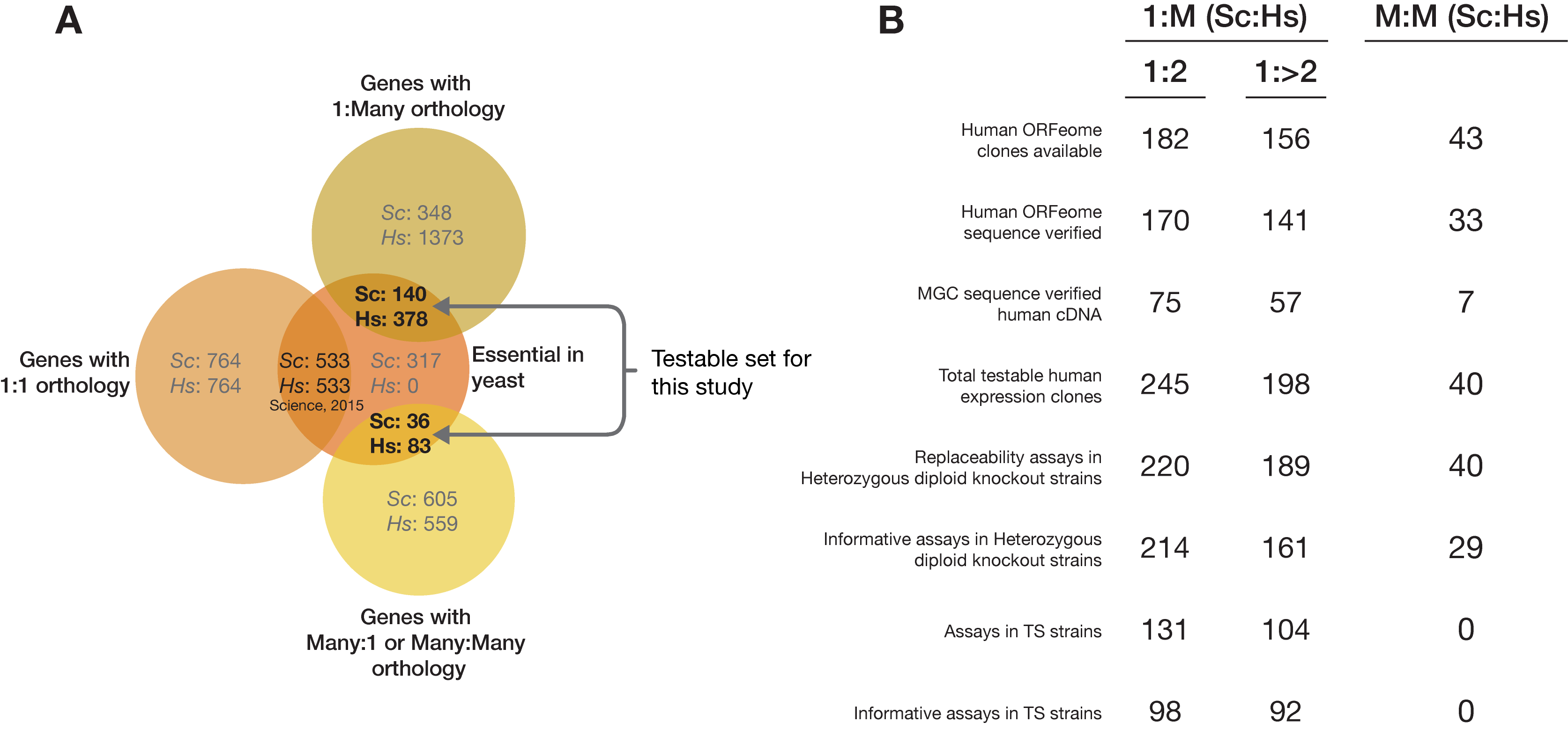

Supplement: S2 Fig — (A) Venn diagram indicating the overlap of essential yeast genes with their human orthologs in various ortholog classes. Ortholog definitions are based on InParanoid [1], whereas essentiality of yeast genes is based on the systematic yeast knockout study by Winzeler and colleagues [22]. (B) A thorough breakdown of the numbers of ortholog pairs identified through to those resulting in informative assays. (TIF) [file pbio.3000627.s002.tif]

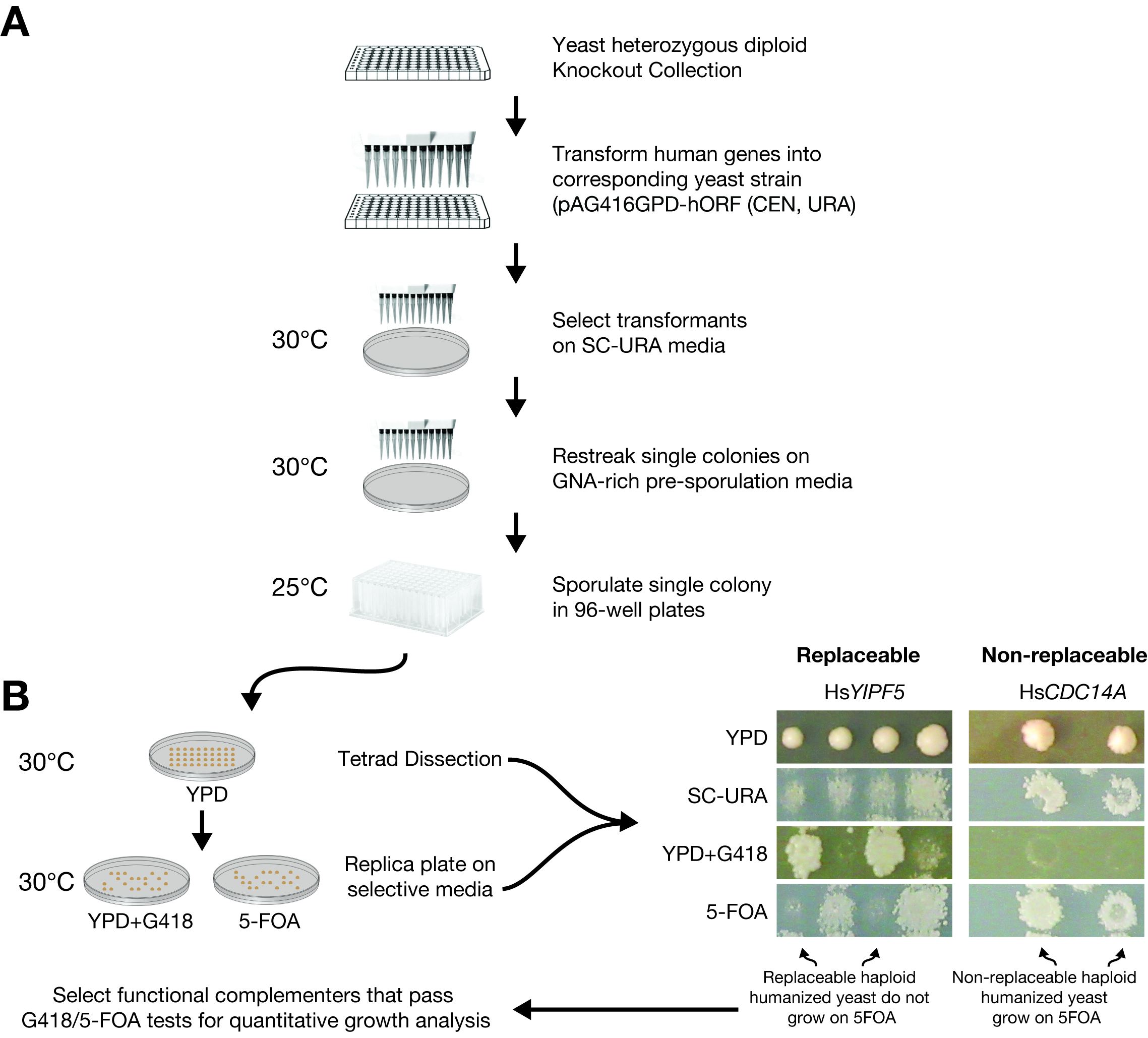

Supplement: S3 Fig — (A) Yeast strains were grown in 96-well plates in selective medium (YPD + 200 μg/ml G418). The matched orthologous human gene expression clones in 96-well plates were transformed into the appropriate yeast strains, followed by selection on the appropriate dropout medium (SD-Ura). The resulting transformants were spotted onto selective medium with appropriate markers to assay complementation. (B) In the case of the yeast hetKO collection, replacement was further verified by carrying out tetrad dissection followed by testing for plasmid dependency (selection on 5-FOA). Representative tetrad dissection assays are shown (right). Hs-YIPF5 functionally replaces its yeast counterpart. Dissected tetrads showed 2:2 segregation, with tetrads 1 and 3 containing the wild-type yeast allele (surviving on 5-FOA), whereas tetrads 2 and 4 contain the KanMX allele (yeast null allele) complemented by the human gene (surviving on YPD + G418). 5-FOA was used for counterselecting the human gene expression vector (CEN6, Ura), whereas YPD + G418 was used to select for the yeast null allele (KanMX selection), respectively. However, in cases on noncomplementers (e.g., Hs-CDC14A), only two yeast spores (carrying the wild-type yeast allele) survive on YPD and fail to grow on YPD + G418 but grow on 5-FOA, indicating plasmid loss. (C) Each confirmed humanized haploid yeast strain was assayed for growth defects to quantify the replaceability. 5-FOA, 5-flouroorotic acid; CDC14A, human dual specificity protein phosphatase CDC14A gene; hetKO, heterozygous diploid knockout, Hs, H. sapiens; SD, synthetic defined; Ura, uracil; YIPF5, human YIPF5 gene; YPD, yeast extract peptone dextrose. (TIF) [file pbio.3000627.s003.tif]

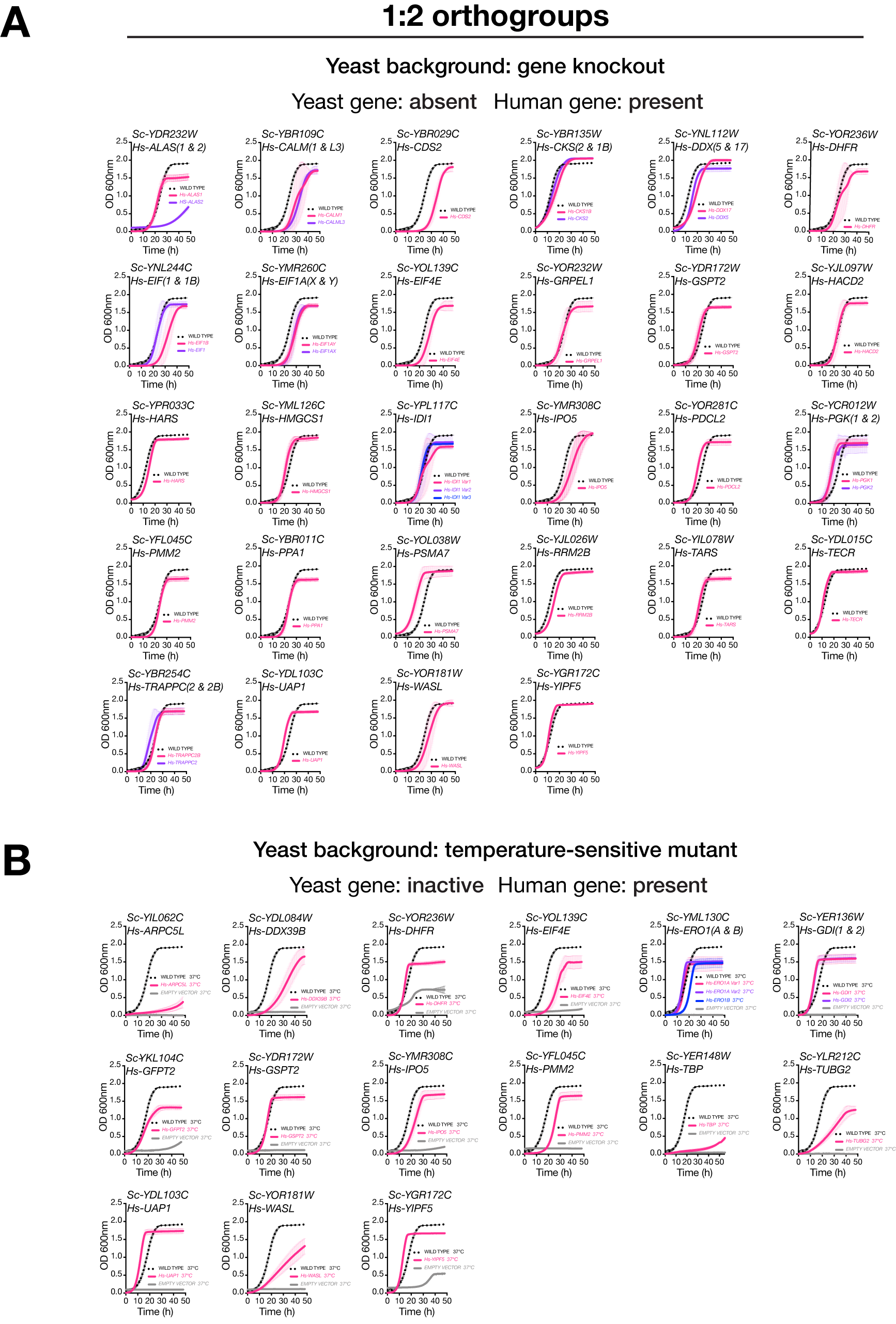

Supplement: S4 Fig — (A) Growth curves of humanized haploid yeast strains obtained after sporulation and tetrad dissection and followed by 5-FOA screening of yeast hetKO strains. The assays were performed in the presence of G418 (yeast gene–absent and human gene–present condition). Haploid yeast gene deletion strains carrying plasmids expressing functionally replacing human genes (colored red or purple solid lines) generally exhibit comparable growth rates to the wild-type parental yeast strain BY4741 (black dotted lines), except in the case of Hs-ALAS2, which shows reduced ability to replace the orthologous yeast gene compared with Hs-ALAS1. Mean and standard deviation plotted from triplicate assays. (B) Growth curves of humanized haploid temperature-sensitive yeast strains performed at a restrictive temperature of 37°C (yeast gene–inactive and human gene–present condition). Temperature-sensitive haploid yeast strains carrying plasmids expressing functionally replacing human genes (colored red, purple, or blue solid lines) generally exhibit comparable growth rates to the wild-type parental yeast strain BY4741 (black dotted lines). The yeast strains harboring empty vector without a corresponding human gene, showing no or poor growth in restrictive temperatures of 37°C (gray solid lines), serve as controls. In total, 208 individual assays were performed (as two independent biological replicates). In all, 45 human genes showed functional replaceability in either assay, whereas 127 did not, and 36 human gene complementation assays were noninformative (cases in which the control experiments did not behave appropriately). Raw data for growth curves are available in S3 Data. 5-FOA, 5-flouroorotic acid; ALAS1, human 5-aminolevulinate synthase ALAS1 gene 1; ALAS2, human 5-aminolevulinate synthase ALAS1 gene 2; hetKO, heterozygous diploid knockout; Hs, H. sapiens. (TIF) [file pbio.3000627.s004.tif]

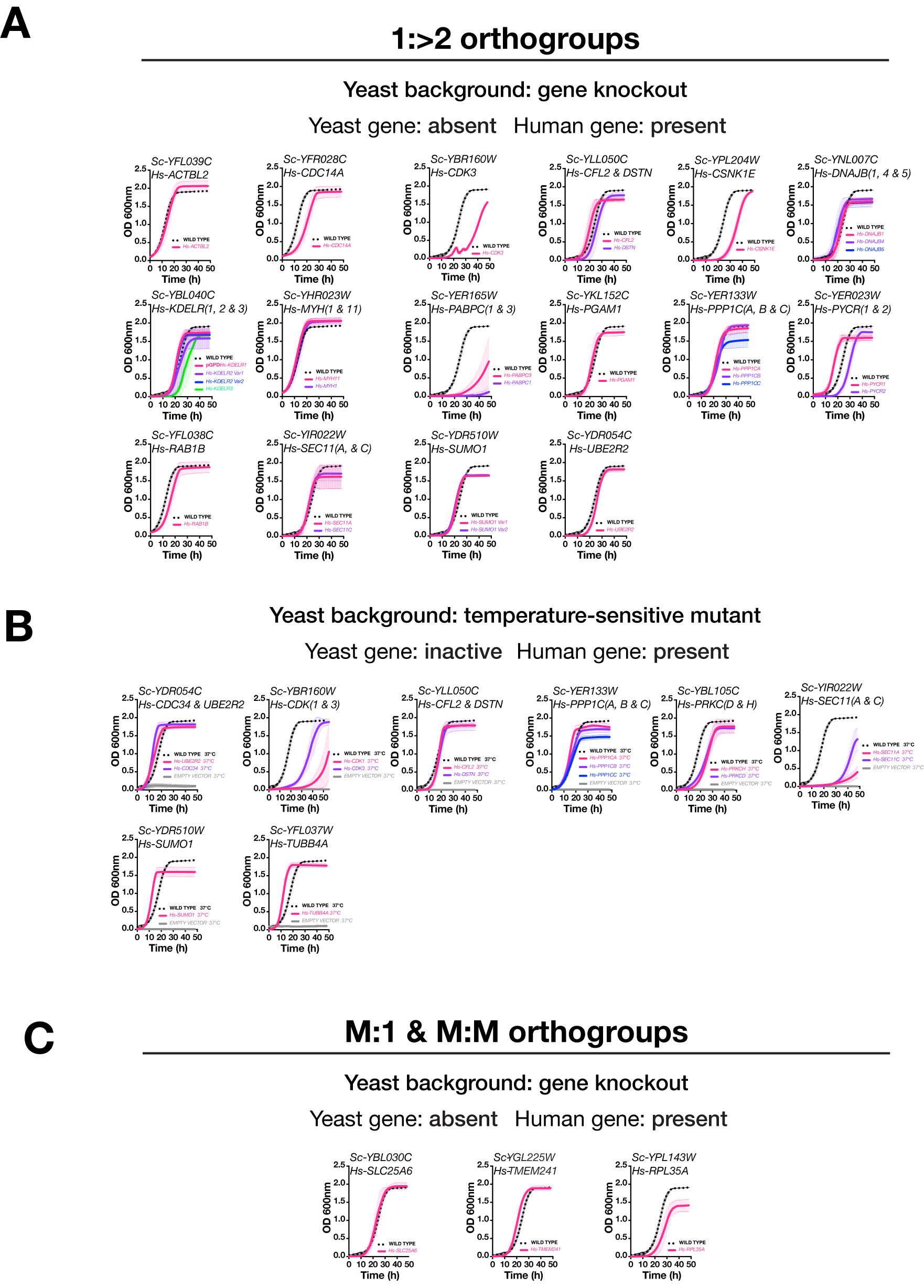

Supplement: S5 Fig — In total, 170 individual assays were performed (as two independent biological replicates): 32 human genes showed functional replaceability in either assay, whereas 105 did not, and 33 human gene replaceability assays were noninformative (cases in which the control experiments did not behave appropriately). (C) Growth curves of humanized haploid yeast strains (M:1 and M:M class) obtained after sporulation and/or tetrad dissection and followed by 5-FOA screening of hetKO strains. The assays were performed in the presence of G418 (yeast gene–absent and human gene–present condition). In total, 40 individual assays were performed (as two independent biological replicates). Three human genes showed functional replaceability, whereas 26 did not, and 11 human gene replaceability assays were noninformative (cases in which the control experiments did not behave appropriately). Mean and standard deviation plotted with N = 3. (A) Growth curves of humanized haploid yeast strains (1:>2 class) obtained after sporulation and/or tetrad dissection followed by 5-FOA screening of hetKO strains. The assays were performed in the presence of G418 (yeast gene–absent and human gene–present condition). Haploid yeast gene deletion strains carrying plasmids expressing functionally replacing human genes (colored red, purple, and blue solid lines) generally exhibit comparable growth rates to the wild-type parental yeast strain BY4741 (black dotted lines), except in the case of Hs-PABPC1 and Hs-PABPC3, which shows reduced ability to replace the orthologous yeast gene. Mean and standard deviation plotted from triplicate assays. (B) Growth curves of humanized haploid temperature-sensitive yeast strains (1:M class) performed at a restrictive temperature of 37°C (yeast gene–inactive and human gene–present condition). Temperature-sensitive haploid yeast strains carrying plasmids expressing functionally replaced human genes (colored red, purple, or blue solid lines) generally exhibit growth rates compa [file pbio.3000627.s005.tif]

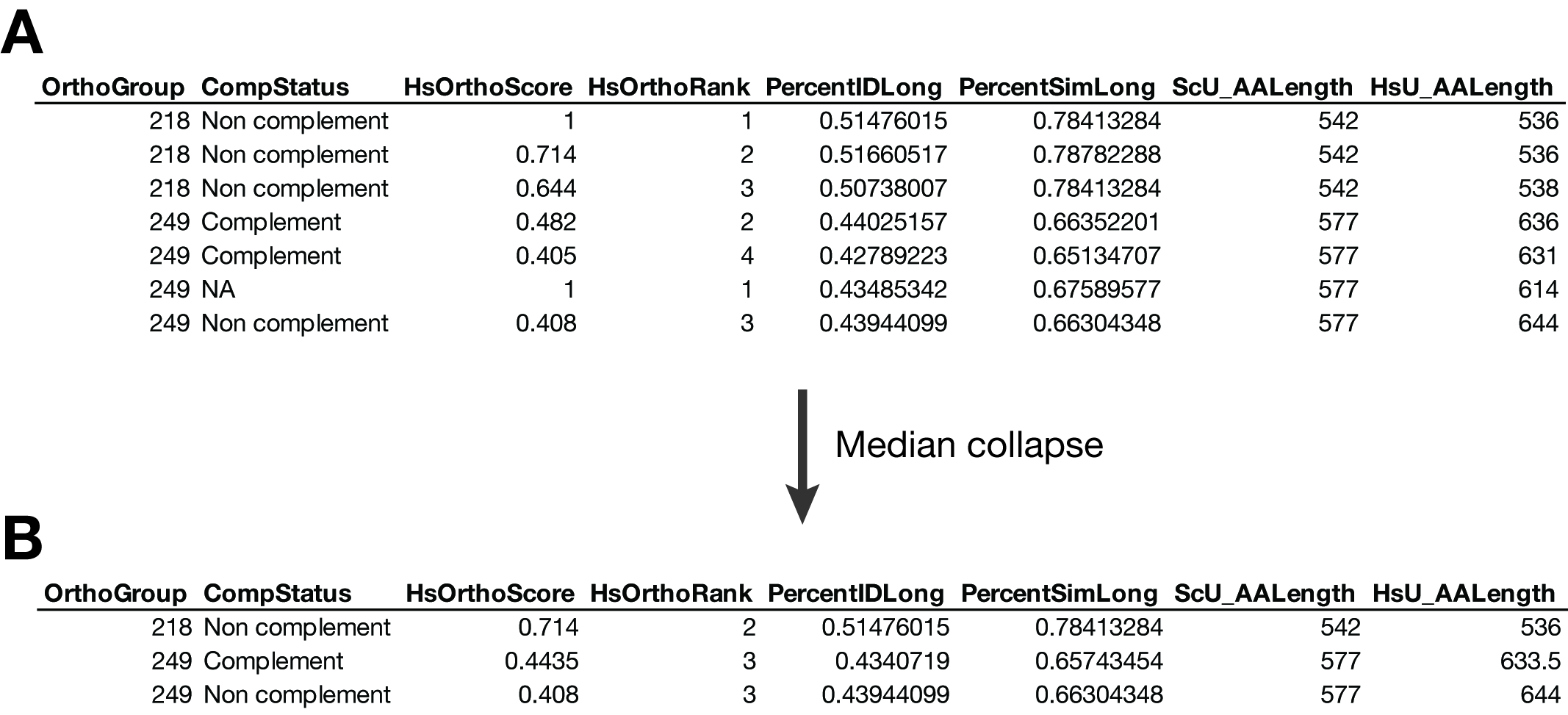

Supplement: S6 Fig — (A) An example subset of full ortholog data for two orthogroups in the 1:M ortholog class. (B) The same data as in (A) following median collapse. Orthogroup 218 has been collapsed to a single metaortholog pair with the status “noncomplement” because all human co-orthologs failed to complement. Group 249 has been collapsed to two metaortholog pairs because there were co-orthologs that complemented and did not. Metaortholog feature values are the medians of the full ortholog values with the same status. 1:M, one-to-many. (TIF) [file pbio.3000627.s006.tif]
